# Dynamics of Microbial Community and Potential Microbial Pollutants in Shopping Malls

**DOI:** 10.1128/msystems.00576-22

**Published:** 2023-01-05

**Authors:** Xin-Li An, Jian-Xin Xu, Mei-Rong Xu, Cai-Xia Zhao, Hu Li, Yong-Guan Zhu, Jian-Qiang Su

**Affiliations:** a Key Laboratory of Urban Environment and Health, Institute of Urban Environment, Chinese Academy of Sciences, Xiamen, China; b State Key Lab of Urban and Regional Ecology, Research Center for Eco-Environmental Sciences, Chinese Academy of Sciences, Beijing, China; c College of Resource and Environmental Science, Fujian Agriculture and Forestry University, Fuzhou, China; Pacific Northwest National Laboratory

**Keywords:** antimicrobial resistance, built environment, human pathogen, microbial assembly, microbial interactions

## Abstract

Shopping malls offer various niches for microbial populations, potentially serving as sources and reservoirs for the spread of microorganisms of public health concern. However, knowledge about the microbiome and the distribution of human pathogens in malls is largely unknown. Here, we examine the microbial community dynamics and genotypes of potential pathogens from floor and escalator surfaces in shopping malls and adjacent road dusts and greenbelt soils. The distribution pattern of microbial communities is driven primarily by habitats and seasons. A significant enrichment of human-associated microbiota in the indoor environment indicates that human interactions with surfaces might be another strong driver for mall microbiomes. Neutral community models suggest that the microbial community assembly is strongly driven by stochastic processes. Distinct performances of microbial taxonomic signatures for environmental classifications indicate the consistent differences of microbial communities of different seasons/habitats and the strong anthropogenic effect on homogenizing microbial communities of shopping malls. Indoor environments harbored higher concentrations of human pathogens than outdoor samples, also carrying a high proportion of antimicrobial resistance-associated multidrug efflux genes and virulence genes. These findings enhanced the understanding of the microbiome in the built environment and the interactions between humans and the built environment, providing a basis for tracking biothreats and communicable diseases and developing sophisticated early warning systems.

**IMPORTANCE** Shopping malls are distinct microbial environments which can facilitate a constant transmission of microorganisms of public health concern between humans and the built environment or between human and human. Despite extensive investigation of the natural environmental microbiome, no comprehensive profile of microbial ecology has been reported in malls. Characterizing microbial distribution, potential pathogens, and antimicrobial resistance will enhance our understanding of how these microbial communities are formed, maintained, and transferred and help establish a baseline for biosurveillance of potential public health threats in malls.

## INTRODUCTION

The built environment is the collection of all manufactured structures, encompassing transportation systems, commercial facilities, and other human-constructed physical surroundings, and represents important human-modified ecosystems with unique microbial assemblages (1). Humans are exposed to colonized bacteria, fungi, and viruses, possibly altering the trajectory of human health. Physical surfaces of buildings are primary ecological sites for microbial adhesion and biofilm formation, and microbial communities are vastly different between various types of built environments and surfaces (2–4). Building occupants contribute significantly to the mall microbiome by releasing human-associated microbes, transmitting outdoor bioparticles, and resuspending microorganisms on the surfaces (4–7). Environmental factors like temperature, geography, meteorology, urbanization, and pets have been associated with the complexity of the microbiome in the built environment (8, 9). Management practices such as routine cleaning and disinfectant usage could lead to unselective removal and killing of microorganisms, thus markedly changing the adaptation strategies (e.g., antimicrobial resistance) of microorganisms and impacting the composition and assembly of microbial residents (10, 11). Advances in high-throughput sequencing have characterized the microbiome in various built environments, including sewer systems (12, 13), hospitals (8, 10), metro systems (14, 15), households (16, 17), and dormitory environments (7, 18). However, patterns of the distribution and dynamics of microbial communities in shopping malls have not yet been reported.

Shopping malls are large enclosed communal gathering places of urban societies with high occupant diversity, densities, and turnovers which people from all walks of life and with different cultures, ages, or physical conditions visit for entertainment, refreshment, and business. Microbial contamination of such a public area could render malls to be a source and reservoir of infections via close interactions between individuals and surfaces (e.g., handrails, floors, and buttons) (14, 15, 19). The interactions could be mediated by microbial transfer between shoes and floor, the exchange of microbes between skin and handles, and the release of gut-related microbes in washrooms (5, 20–22). Although there is a paucity of evidence to manifest direct transfer of microorganisms from built environments to humans, some investigations have observed the potential transmission of pathogens and antimicrobial resistance to humans through surfaces and equipments (7, 23). Several pioneering studies for the mall microbiome have detected high bacterial densities on tables, trays, and cleaning cloths in food courts (23, 24). Potential pathogens such as Staphylococcus aureus, Pseudomonas spp., and Gram-negative bacilli have been isolated from shopping malls, and more than 50% of samples were detected with bacterial contamination (25, 26). Multidrug-resistant pathogenic species, including *Stenotrophomonas*, *Aeromonas*, Acinetobacter, Pseudomonas, and *Bacillus*, were detected from escalator handrails, lift buttons, and shopping carts by culture-based methods (11, 27). Furthermore, recent advances on the coronavirus pandemic have confirmed that shopping malls were associated with locally transmitted cases of COVID-19 and were identified as one of the superspreading environments for COVID-19 (28–30). These findings suggest that exposure to the mall microbiome might contribute to health burdens of human infectious diseases (15, 31).

Antimicrobial resistance (AMR) is a global health threat that has resulted in high mortality and health care costs. Research on hospitals and metro systems has confirmed that contaminated surfaces or fomites contribute to the spread of bacterial infections and AMR (14, 29, 31, 32). *Enterobacteriaceae* comprise a number of pathogens, such as Klebsiella, Enterobacter, Escherichia coli, Salmonella, and *Citrobacter*, and are commonly used as an indicator of hygiene and contamination in environments (33). The emergence and spread of AMR in *Enterobacteriaceae* complicate the treatment of infectious diseases. The World Health Organization (WHO) has prioritized carbapenem-resistant *Enterobacteriaceae* as a top-priority pathogens for an urgent need to develop new antibiotics (34). Available evidence has demonstrated that exposure to disinfectants and cleaning agents in built environments poses a possible risk of AMR development of *Enterobacteriaceae* species (35–37), and the environmental exposures of built environments may contribute to the skin microbiome and resistome (29, 31). Therefore, deciphering AMR of *Enterobacteriaceae* species is crucial for better understanding the transmission and evolution of AMR and pathogens in built environments.

In the present study, microbial communities, together with human pathogens and their antimicrobial resistance, were characterized from 20 populated shopping malls as well as the surrounding road dust and greenbelt soil in spring and summer. We hypothesized that microbial communities exhibit distinct season and habitat specificity and that microbial community assembly is driven mainly by stochastic processes owing to regular cleaning and disinfection in malls. We further predicted that human and outdoor (road dust and greenbelt soil) microbiomes contribute largely to the mall microbiome via direct or indirect transmission, and thus mall surfaces harbor more potential human pathogens and AMR determinants. To test these hypotheses, bacterial 16S rRNA gene and fungal internal transcribed spacer (ITS) amplicon sequencing was conducted to characterize the microbial community structures and compositions. Neutral community models (NCM), SourceTracker models, and random forest models were employed to depict the microbial community assembly processes, identify the microbial signatures related to environmental preference, and track the potential microbial source of shopping malls. High-throughput quantitative PCR (HT-qPCR) assays for human pathogens and genomic analysis of *Enterobacteriaceae* isolates were further performed to determine the pathogen distribution and the AMR and virulence gene burdens.

## RESULTS

### Microbial community diversity and composition.

**FIG 1:**
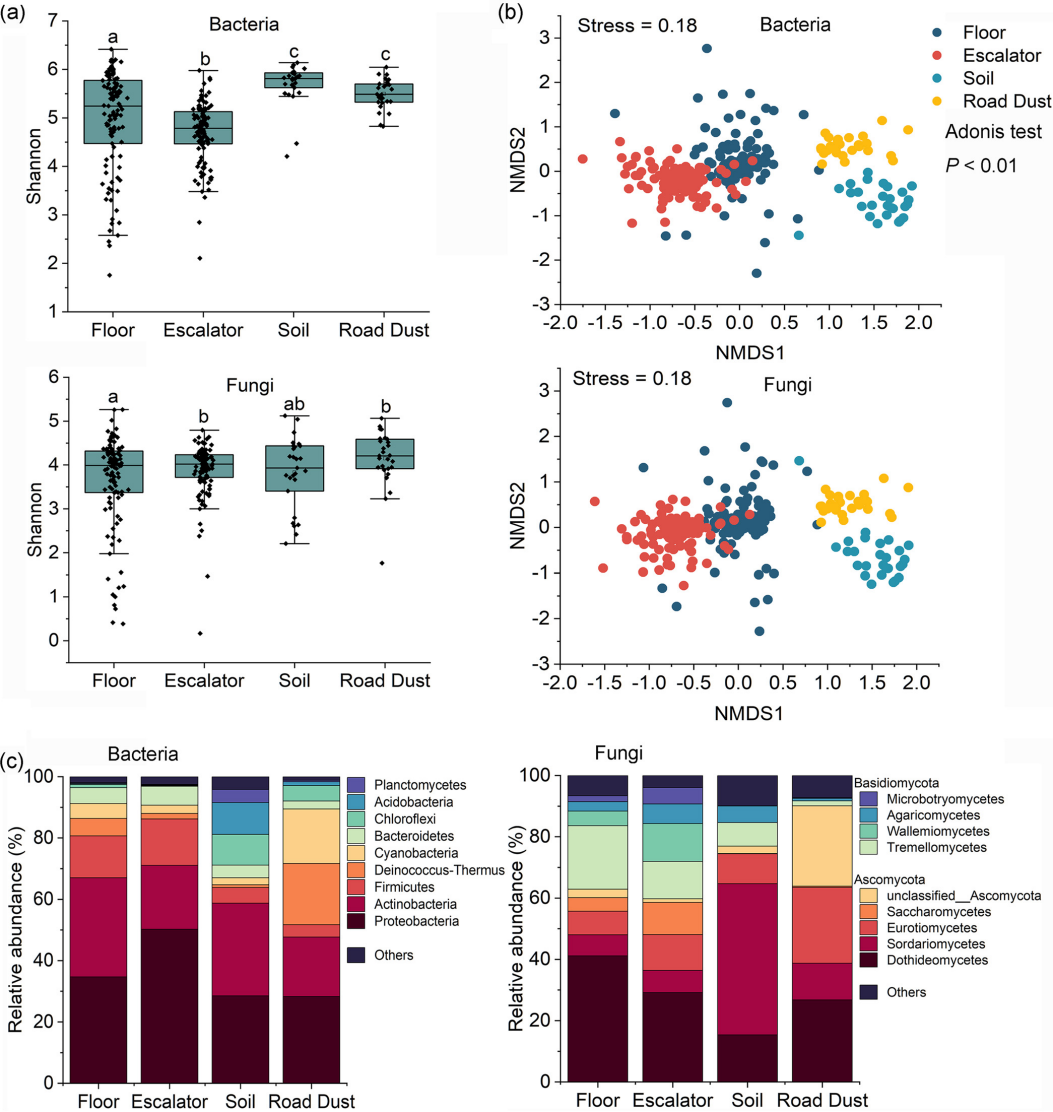
Diversity and composition of the floor, escalator, greenbelt soil, and road dust microbial communities. (a) Shannon diversity of bacterial and fungal communities across different inhabitants. (b) Distribution patterns of microbial communities visualized using NMDS analysis based on Bray-Curtis distance. (c) Relative abundances of bacterial taxa (at the phylum level) and fungal taxa (at the class level).

Microbial community diversity and composition were evaluated by amplicon sequence analysis of 274 DNA samples obtained from floor surfaces, escalator surfaces, road dusts, and greenbelt soils (see Table S1 in the supplemental material). In total, 25,806 bacterial amplicon sequence variants (ASVs) and 19,656 fungal ASVs were assigned, and floor surfaces had the highest number of ASVs, followed by escalators, road dusts, and greenbelt soils. There were 1,276 shared bacterial ASVs observed in four habitats, while shared fungal ASVs were not found in any of the four habitats. The bacterial community of floor surfaces had the highest species richness (Chao 1), followed by escalators, road dusts, and greenbelt soils (Fig. S1a). However, significantly higher diversities (Shannon and Simpson diversities) were found in the bacterial communities of greenbelt soils and road dusts than of floors and escalators (*P < *0.05) (Fig. 1a; Fig. S1b). For the alpha-diversity of the fungal community, escalators had the lowest ASV number, and road dust harbored the highest number of ASVs (*P < *0.05) (Fig. S1c). The fungal community of floor surfaces showed significantly lower Shannon and Simpson index values than the others (*P < *0.05) (Fig. 1a; Fig. S1d). Significant seasonal variation in bacterial or fungal alpha-diversity was not observed (*P > *0.05), while both bacterial and fungal communities were significantly grouped with habitats (Adonis test, *P < *0.001) and seasons (Adonis test, *P < *0.001) (Fig. 1b; Fig. S1e and f). Clustering of microbial communities from floors or escalators was not observed across malls (Fig. S2).

10.1128/msystems.00576-22.1 FIG S1 Diversity and composition of floor, escalator, soil, and road dust microbial communities. (a and b) Chao 1 index and Simpson index of bacterial communities. (c and d) Chao 1 index and Simpson index of fungal communities. Different letters indicate significant differences among different samples (mean ± standard deviation [SD]; ANOVA, *P* < 0.05). NMDS analysis revealed significant seasonal differences in floor (e) and escalator (f) microbial communities (Adonis test, *P < *0.01). (g and h) Bacterial (at the phylum level) and fungal (at the class level) community compositions varied with seasons (spring and summer). Download FIG S1, TIF file, 1.3 MB. Copyright © 2022 An et al. 2022 An et al. https://creativecommons.org/licenses/by/4.0/ This content is distributed under the terms of the Creative Commons Attribution 4.0 International license.

10.1128/msystems.00576-22.2 FIG S2 Principle-coordinate analysis based on Bray-Curtis distance showed that the microbial community (bacterial community [a and b] and fungal community [c and d]) from the surfaces of floors or escalators was not grouped across shopping malls. Download FIG S2, TIF file, 0.6 MB. Copyright © 2022 An et al. 2022 An et al. https://creativecommons.org/licenses/by/4.0/ This content is distributed under the terms of the Creative Commons Attribution 4.0 International license.

10.1128/msystems.00576-22.7 TABLE S1 Summary for the collected samples. Download Table S1, DOCX file, 0.1 MB. Copyright © 2022 An et al. 2022 An et al. https://creativecommons.org/licenses/by/4.0/ This content is distributed under the terms of the Creative Commons Attribution 4.0 International license.

*Proteobacteria* and *Actinobacteria* were the predominant bacterial phyla across all samples, occupying 46.0% to 70.5% of bacterial communities (Fig. 1c; Fig. S1g). *Firmicutes* was the third most abundant phylum on floor and escalator surfaces. Greenbelt soils harbored more abundant *Chloroflexi* and *Acidobacteria* (*P < *0.05) and road dusts harbored more *Cyanobacteria* and *Deinococcus*-*Thermus* (*P < *0.05) than did the other habitats. A total of 14 fungal phyla were detected among all samples, and the most abundant phyla were *Ascomycota* and *Basidiomycota* (Fig. 1c), in which *Dothideomycetes* was the most abundant class of bacteria in floor, escalator, and road dust samples, representing 26.8% to 41.1% of the overall fungal communities, while *Sordariomycetes* was the dominant class in greenbelt soils. Seasonal variations in fungal community composition were also observed in all samples. Summer samples were significantly dominated by *Eurotiomycetes*, *Saccharomycetes*, and *Wallemiomycetes*, and spring samples mainly harbored *Tremellomycetes*, *Agaricomycetes*, *Microbotryomycetes*, and *Dothideomycetes* (one-way analysis of variance [ANOVA] tests, *P < *0.05) (Fig. S1h).

### Determination of core microbial communities.

We defined the core microbial communities as taxa detected in more than 80% of samples according to previous studies (12, 38). The core bacterial communities contained 36 bacterial ASVs, accounting for 3.9% to 19.9% of bacterial sequences. These core bacterial species were not uniformly abundant across all sample types. The most abundant core bacterial ASVs were ASV5031_g_Acinetobacter (0.9%), ASV4883_g_*Enhydrobacter* (0.84%), and ASV18928_g_*Kocuria* (0.82%), in which Acinetobacter baumannii and Kocuria kristinae were associated with infectious diseases. The core fungal community contained only one ASV (ITS_745_g_*Cladosporium*, commonly causing allergies and asthma), accounting for 5.6% of the total fungal sequences and occurring exclusively on escalators (8.7%) and floors (5.2%). The shared microbial communities presenting in all samples (100% of samples) of each habitat were also determined. A majority of the shared bacterial and fungal ASVs in soils were also observed in the shared microbial communities of road dusts. In contrast, none of shared microbial ASVs observed in soils or road dusts was found in escalator or floor microbial communities and vice versa.

### Microbial interaction network and community assembly.

A maximum number of significant (*P < *0.05) bacterial pairwise correlations was observed for floors (12,621), followed by those for escalators (9,837), road dusts (6,435), and soils (4,374) (Fig. S3). In the fungal community, floors harbored the maximum number of significant pairwise relationships (5,961), followed by soils (4,480), road dusts (4,480), and escalators (4,414). Among these microbial associations, the number of positive correlations was significantly higher than the negative ones (Table S2). Topological features of cooccurrence networks based on significant (*P < *0.01) and robust (Pearson’s *r *= 1) microbial correlations showed more complex interactions in bacterial communities than fungal communities, and the networks were more dense in road dust and soil samples than in escalator and floor samples.

10.1128/msystems.00576-22.3 FIG S3 Correlation network analysis depicted the microbial interactions in different habitats. A connection represents a strong (Pearson’s *r *= 1) and significant (*P < *0.01) correlation. Nodes indicate the taxonomic affiliation at the family level. Node size is proportional to the number of connections. Download FIG S3, TIF file, 2.5 MB. Copyright © 2022 An et al. 2022 An et al. https://creativecommons.org/licenses/by/4.0/ This content is distributed under the terms of the Creative Commons Attribution 4.0 International license.

10.1128/msystems.00576-22.8 TABLE S2 Topological features of cooccurrence networks for microbial communities. Download Table S2, DOCX file, 0.04 MB. Copyright © 2022 An et al. 2022 An et al. https://creativecommons.org/licenses/by/4.0/ This content is distributed under the terms of the Creative Commons Attribution 4.0 International license.

**FIG 2:**
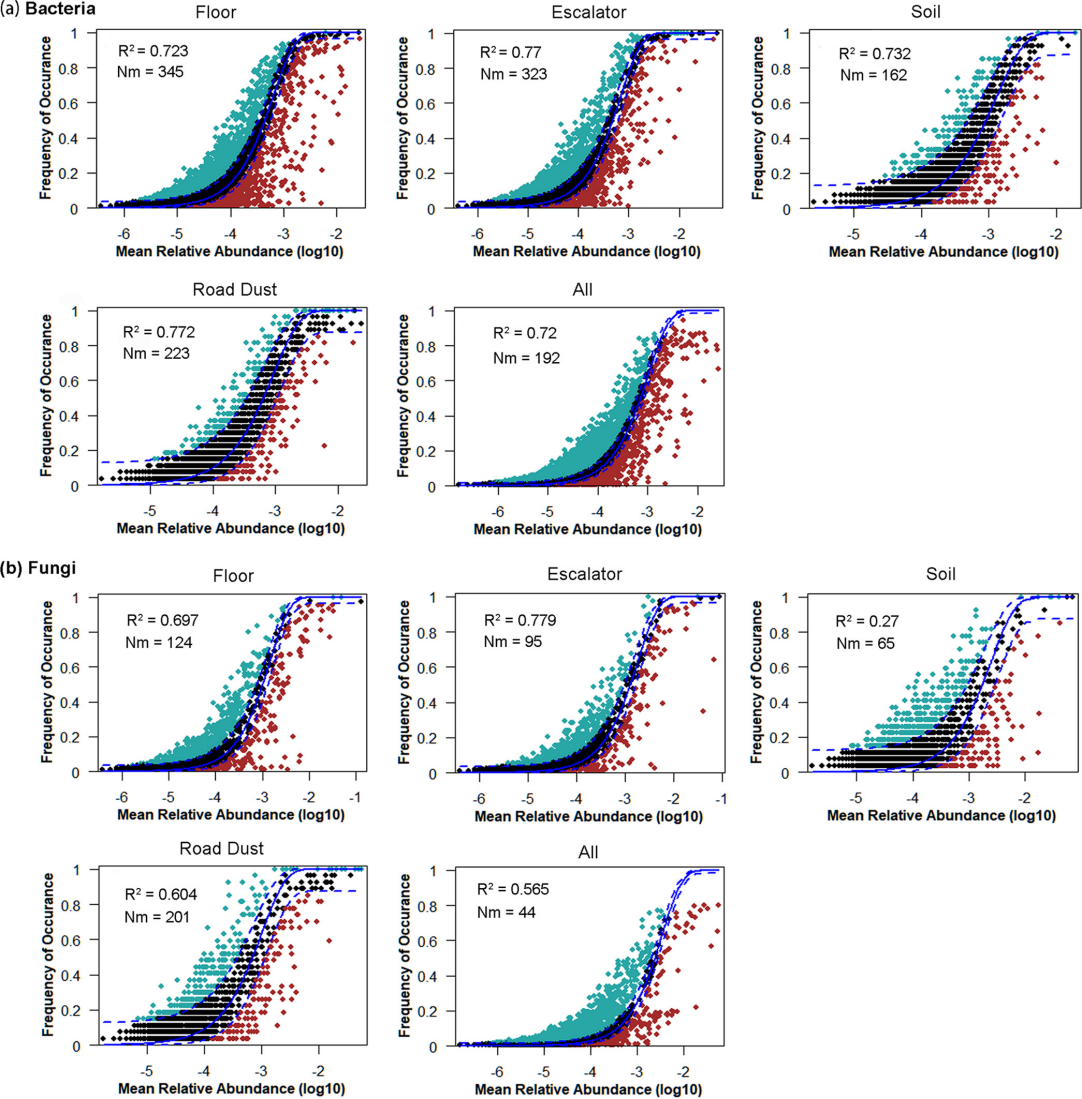
NCM of bacterial (a) and fungal (b) communities across floor, escalator, soil, and road dust. “All” represents microbial communities from all habitats. Dark dots indicate occurrence frequency within the 95% confidence interval (dashed blue lines). ASVs that occur more and less frequently than predicted by NCM are marked in green and red, respectively. The coefficient of determination (*R*^2^) is the goodness of fit of the neutral model, and it ranges from 0 (no fit) to 1 (perfect fit). *Nm* indicates the estimates of the metacommunity size times immigration rate. *N* represents the metacommunity size, and *m* is the immigration rate.

To further explore the community assembly process controlling microbial diversity patterns, we applied the NCMs to characterize the relationship between the predicted ASV occurrence frequencies and their relative abundances (Fig. 2). The best-fit neutral model revealed that the models explained 70% to 77.2% of bacterial community variances for each habitat and overall samples. The NCMs of fungal communities also explained large proportions of variation for floor (69.7%), escalator (77.9%), road dust (60.4%), and overall (56.5%) samples, while the NCM for the soil fungal community explained only 27% of the variance. The values of both *Nm* (estimate of dispersal between communities) and *m* (immigration rate) of the bacterial communities exceeded those of the fungal communities, where soil fungal communities were observed to have the lowest species dispersal (*Nm *= 65) and immigration rate (*m *= 0.14) (Fig. S4a). The normalized stochasticity ratio (NST) index was also calculated to evaluate the relative importance of deterministic (<50%) and stochastic (>50%) community assembly processes. NSTs of both bacterial and fungal communities were substantially above the 50% boundary (Wilcoxon test, *P < *0.001), except that those of the floor bacterial community (56%) and soil fungal community (52.5%) were close to the margin, indicating the significant role of stochastic processes in microbial assembly (Fig. S4b).

10.1128/msystems.00576-22.4 FIG S4 (a) The immigration rates at the community level were predicted by the neutral community model. Immigration rates indicate the probability that a dead individual is replaced by an immigrant. (b) The normalized stochasticity ratio (NST) index evaluated the relative importance of deterministic (<50%) and stochastic (>50%) community assembly processes. Download FIG S4, TIF file, 0.5 MB. Copyright © 2022 An et al. 2022 An et al. https://creativecommons.org/licenses/by/4.0/ This content is distributed under the terms of the Creative Commons Attribution 4.0 International license.

### Identification of microbial signatures.

**FIG 3:**
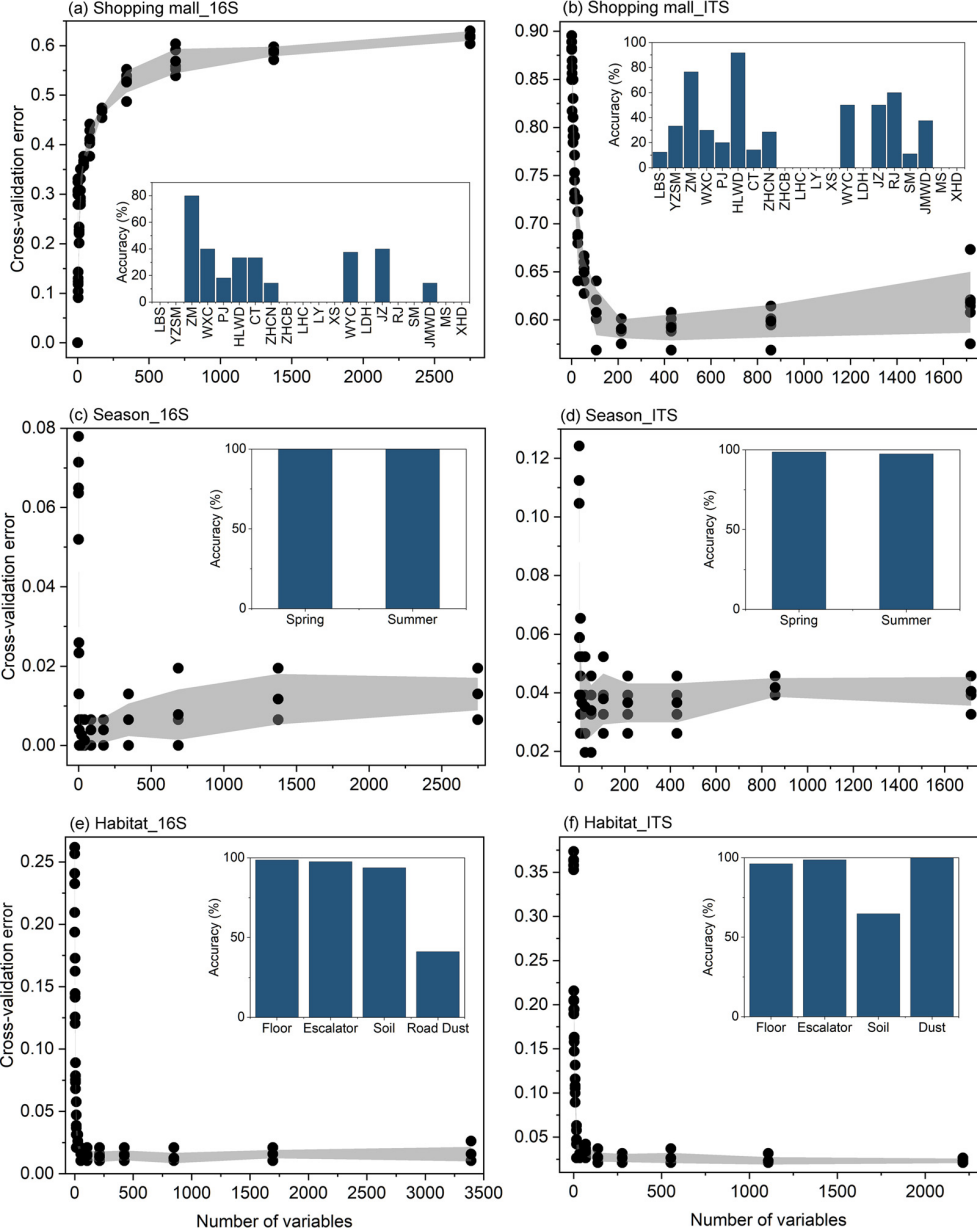
Classification accuracy of the optimized random forest models for assigning samples to shopping malls, seasons, and habitats. The top 10 important bacterial and fungal signatures were selected as the optimal biomarker sets to optimize the random forest model based on the five cross-validation sets of trained samples. Embedded histograms revealed that the prediction performance of random forest models was evaluated using the testing set (validation set), measured as accuracy.

To determine the predictive potential of microbial fingerprints for shopping mall, season, and habitat discrimination, we trained the random forest classifiers (RFCs) for microbial communities and evaluated their performance in differentiating samples with their correct origins based on a 10-fold cross-validation framework (Fig. 3; Fig. S5). The trained RFCs were highly sensitive and specific for determining from which seasons or habitats a sample was taken, which indicates consistent differentiation of microbial communities from different seasons or habitats (Table S3). However, microbial community-based identification of individual malls showed high classification error ratios (68.83% of out-of-bag (OOB) error rate of bacterial community; 64.05% of out-of-bag error rate of fungal community). This is likely due to the presence of the core microbiomes in the floor and escalator microbial communities from shopping malls. Moreover, the sample size in each shopping mall also affected the accuracy of RFCs. Based on the MeanDecreaseAccuracy and MeanDecreaseGini metrics, the most predictive taxonomic signatures for season and habitat classifications were evaluated by the trained RF models. The top 10 ASVs/ITS sequences with the highest feature importance scores were mostly affiliated with the bacterial phyla *Actinobacteria* and *Proteobacteria* or the fungal phyla *Ascomycota* and *Basidiomycota*.

10.1128/msystems.00576-22.5 FIG S5 A random forest model indicates the predictor importance (percentage of increase in mean square error) of dominant ASVs (>274 in total number) as drivers for microbial community in the whole profile. The accuracy importance measure was computed for each tree and averaged over the forest (1,000 trees). Percentage increases in the mean squared error (MSE) of variables were used to estimate the importance of these predictors, and higher MSE% values imply more important predictors. The embedded charts showed that the true-positive percentage and the false-positive percentage achieved an area under the curve (AUC) value of 0.9024 with s 95% confidence interval (CI) of 0.8168 to 0.9669 between trained (*n *= 183) and tested (*n *= 91) samples in the validation cohort (*P < *0.0001). Download FIG S5, TIF file, 2.0 MB. Copyright © 2022 An et al. 2022 An et al. https://creativecommons.org/licenses/by/4.0/ This content is distributed under the terms of the Creative Commons Attribution 4.0 International license.

10.1128/msystems.00576-22.9 TABLE S3 Summary of predictive accuracy of random forest supervised learning models for habitat, season, and shopping mall classification. Download Table S3, DOCX file, 0.05 MB. Copyright © 2022 An et al. 2022 An et al. https://creativecommons.org/licenses/by/4.0/ This content is distributed under the terms of the Creative Commons Attribution 4.0 International license.

### Tracking human microbial footprint in built environments.

Since occupants and outdoor environments are the two main sources of microbes in built environments, we evaluated the potential contribution of their microbial communities to the indoor microbiomes (floors and escalators) of shopping malls by using Bayesian-based source tracking models (Fig. S6). SourceTracker analysis revealed that human palms were a major source of escalator microbiota, approximately 60% of which was associated with human palms. Bacterial taxa associated with air, road dust, and soil were predicted to have a minor influence on the escalator community, accounting for 1% to 2.5% of escalator microbes. Similarly, floor microbiota contained a high proportion of ASVs with human palm (~ 25%) and road dust (15%) sources, while air (5.5%) and soil (2%) contributed a small percentage of the microbial composition of floors. However, a considerable proportion of floor ASVs were predicted with unknown sources.

10.1128/msystems.00576-22.6 FIG S6 SourceTracker models predicted the proportions of bacterial taxa from floors and escalators that come from possible sink environments. The bacterial communities from soils, road dust, air, and human palms were used as sources. Download FIG S6, TIF file, 0.1 MB. Copyright © 2022 An et al. 2022 An et al. https://creativecommons.org/licenses/by/4.0/ This content is distributed under the terms of the Creative Commons Attribution 4.0 International license.

### Prevalence of potential human pathogens and antimicrobial resistance.

**FIG 4:**
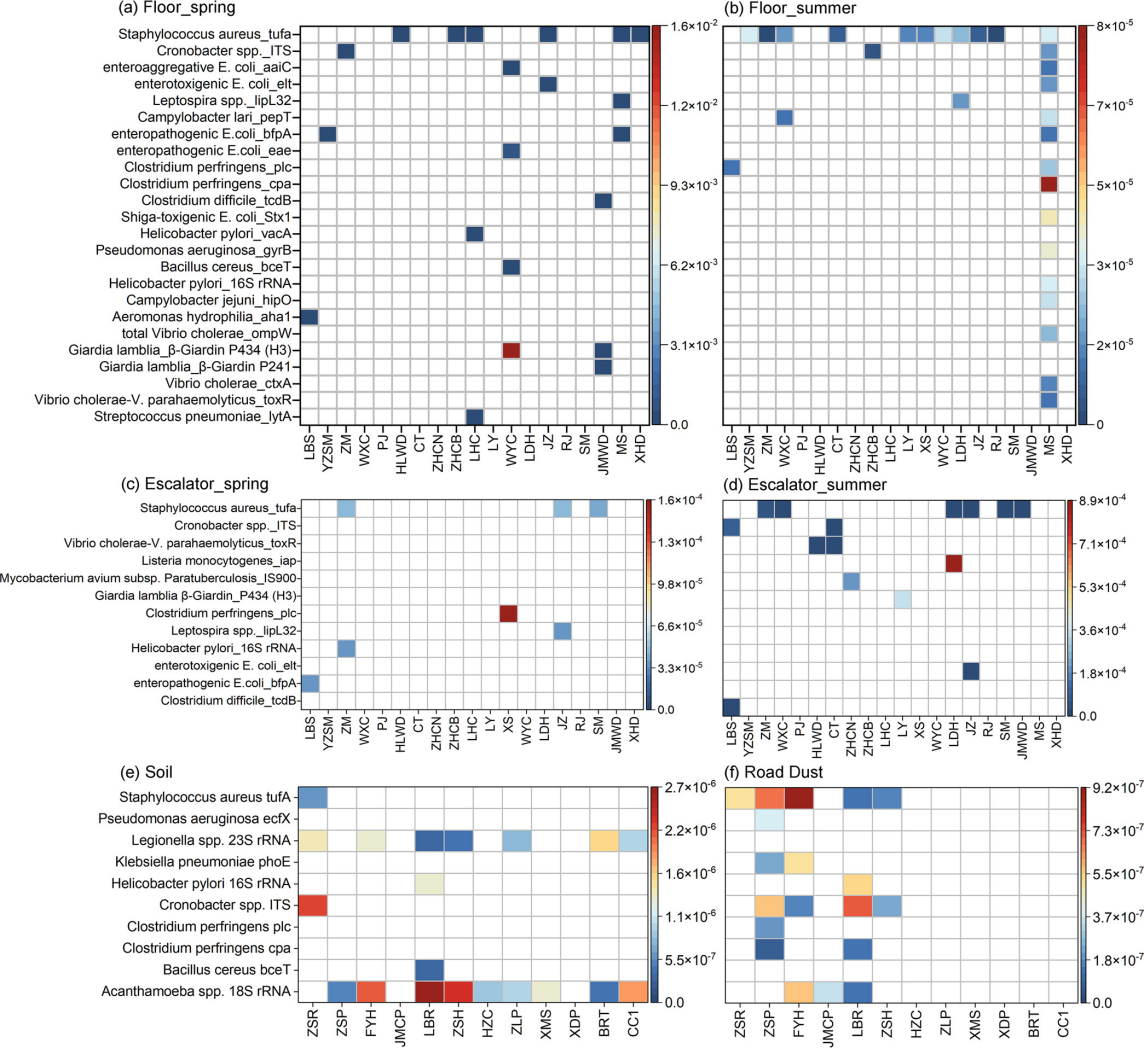
Heat map showing the incidence and relative abundance (copies/copy of 16S rRNA gene) of marker genes for human pathogens detected from floors (a and b), escalators (c and d), soils (e), and road dusts (f) by using HT-qPCR assays.

HT-qPCR assays showed that a highest relative abundance of pathogens was observed for floors (6.0 × 10^−7^ to 8.3 × 10^−2^ copies/copy of 16S rRNA), followed by escalators (3.9 × 10^−4^ to 4.5 × 10^−3^ copies/copy of 16S rRNA), soils (2.3 × 10^−6^ to 2.4 × 10^−5^ copies/copy of 16S rRNA), and road dusts (1.8 × 10^−6^ to 1.1 × 10^−5^ copies/copy of 16S rRNA) (Fig. 4). Seasonal variations in the distribution of marker genes for pathogens were observed. For examples, floors in the spring harbored more abundant marker genes than floors in the summer, and for escalators, marker genes were more abundant in summer than in spring. Staphylococcus aureus was the most prevalent pathogen, with varied detection frequencies for floors (75%), escalators (25%), and road dusts (42%). *Acanthamoeba* spp., which was the most frequently detected pathogen in soils, with 75% of sites positive, is commonly found in natural environments and responsible for a fatal encephalitis and keratitis in humans.

**FIG 5:**
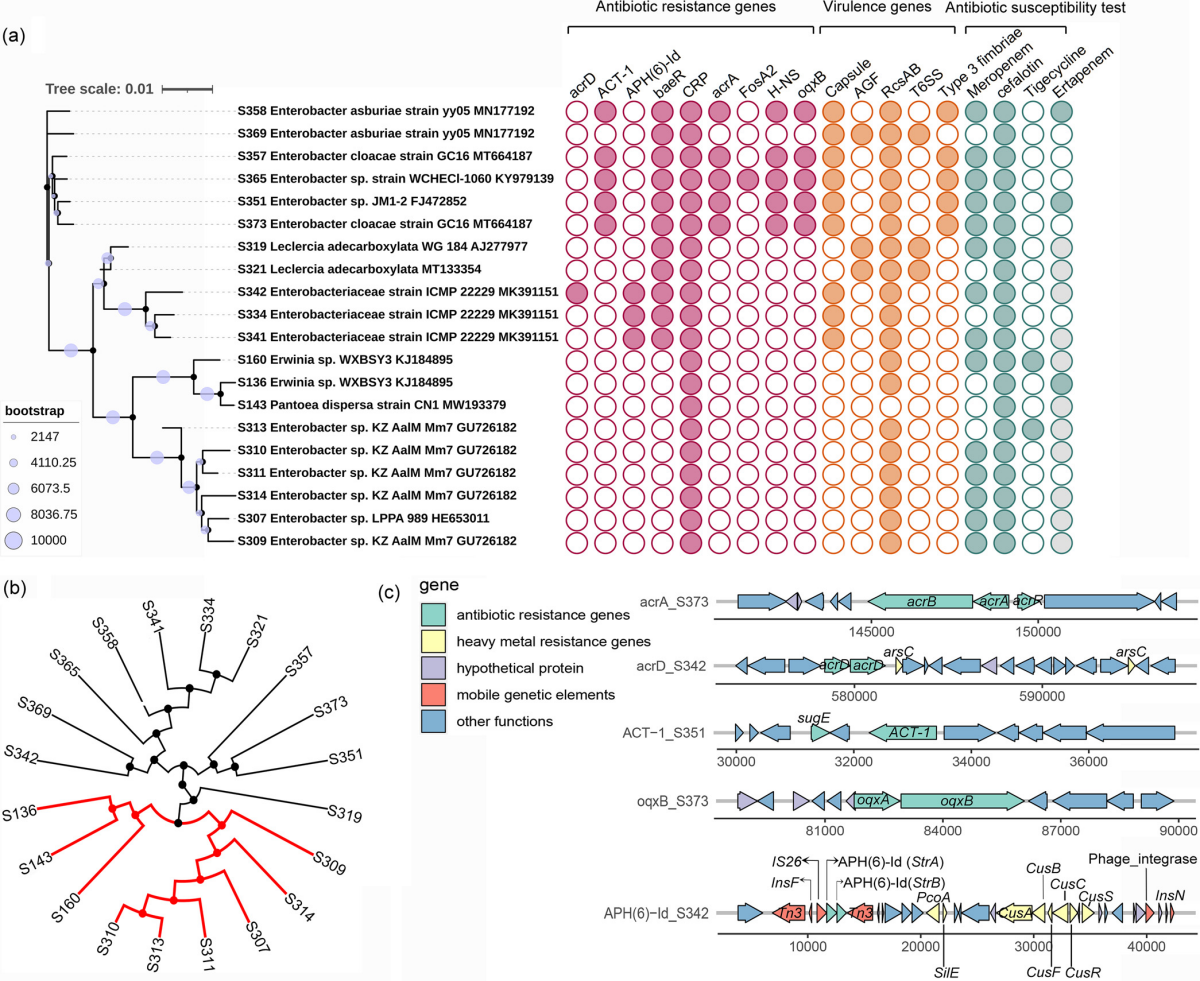
Genomic analysis and antimicrobial susceptibility of *Enterobacteriaceae* species (*n *= 20) retrieved from floors and escalators. (a) Phylogenetic tree (left) of all strains based on full-length 16S rRNA gene sequences (bootstrap = 10,000). A bubble plot (right) reflects the detection of antibiotic resistance genes (annotated by searching against CARD database; red) and virulence genes (annotated by searching against VFDB database; orange) from genome sequences and antimicrobial susceptibility test (green; strains marked in gray are the ones for which the ertapenem susceptibility test was not performed). (b) A rooted maximum-likelihood phylogenetic tree was constructed based on the alignment of the core genome single nucleotide polymorphisms. The strains in the red branches clustered together, which was consistent with the clustering of their full-length 16S rRNA genes. (c) Flanking regions of the detected antibiotic resistance genes in contigs. Arrows indicate the direction of gene transcription. Different genes are indicated by different colors. Genes with ≥98% amino acid identity and a query coverage of ≥99% were annotated by mapping sequences against the NCBI nr database. AGF, curli fibers/thin aggregative fimbriae.

A total of 302 bacterial strains were isolated from CHROMagar *Escherichia coli* and coliforms plates, and 20 strains were affiliated with the family *Enterobacteriaceae*, all of which were retrieved from floors (Fig. 5a). Core genome single nucleotide polymorphism (SNP)-based phylogenetic analysis revealed a clustering profile similar to that of the 16S rRNA-based phylogenetic tree (Fig. 5b). A majority of strains were resistant to cephalothin (19/20) and meropenem (15/20), and a few of the isolates were resistant to tigecycline (2/20) and ertapenem (3/10) (Fig. 5a). All strains carried the *CRP* gene (ARO:3000518), which encodes a regulator associated with the expression of the MdtEF multidrug efflux pump and is responsible for fluoroquinolone, macrolide, and penam resistance. Strains S358 (Enterobacter asburiae), S357 (Enterobacter cloacae), S365 (Enterobacter sp.), S351 (Enterobacter sp.), and S373 (Enterobacter cloacae) harbored the same antibiotic resistance genes (ARGs), including *ACT-1* (beta-lactamase conferring resistance to carbapenem, cephalosporin, cephamycin and penam; ARO:3001821), *baeR* (a regulator for the expression of MdtABC and AcrD efflux pump complexes encoding resistance to aminocoumarin and aminoglycoside; ARO:3000828), *acrA* (AcrAB-TolC multidrug efflux for triclosan, cephalosporin, and fluoroquinolone; ARO:3004042), the H-NS gene (a regulator for the resistance-nodulation-division [RND]-type multidrug exporters for cephalosporin, cephamycin, fluoroquinolone, and tetracycline; ARO:3000676), and *oqxB* (RND efflux pump conferring resistance to fluoroquinolone, glycylcycline and tetracycline, ARO: 3003923). Discrepancies between antimicrobial resistance phenotyping and genotyping were also observed in some isolates. For example, tigecycline resistance-related *tet* gene variants were not found, although the resistance phenotype was detected in isolates S160 and S313. We further analyzed the accessory genes of several ARGs and found that the *ACT-1*-carrying isolates possessed the same genomic backbone comprising a resistance gene cluster, *sugE* (quaternary ammonium compound-resistance protein)–*orf*–*ACT-1* (Fig. 5c). The contigs carrying *APH(6)-Id* harbored complex genetic contexts, including transposon, ARGs, and copper resistance genes (Tn*3*-*insF*-IS*26*-*strA*-*strB*-Tn3 and *cusA*-*cusB*-*cusC*-*cusR*-*orf*-*cusS*-*orf*-*orf*-*orf*-*orf*-*orf*-phage_integrase-*orf*-*orf*-*insN*). The main resistance mechanisms of these detected ARGs were multidrug efflux systems and antibiotic target alteration. For virulence-associated genes (VAGs), *rcsAB* was frequently detected in these isolates (19/20, 95%), which is related to the regulation of VAGs. Other VAGs encoding capsule (antiphagocytosis), AGF (curli fibers/thin aggregative fimbriae), T6SS (secretion system), and type 3 fimbriae (mediating biofilm formation) were found with detection frequencies ranging from 15% to 45%.

## DISCUSSION

Shopping malls represent the main source of microbial exposures for human occupants, providing a unique venue for microbial interactions and exerting direct or indirect effects on human living space and health. Our study characterized the microbial community profiles, community assembly mechanisms, and potential pathogens of the floors and escalators of shopping malls and their surrounding road dusts and greenbelt soils. These habitats represent distinct ecological niches with homogeneous dispersals (community evenness) of species, various genetic pools (species richness), and different community compositions. Homogeneous dispersal of bacterial species dominated within road dusts and greenbelt soils, while floors and escalators might have a large genetic pool of microorganisms. It was presumed that soil and dust legacy possibly affected community evenness, and the infiltration of outdoor air/dust or biological particles from cleaning processes being settled contributed to the microbial richness of mall surfaces. The fungal alpha-diversity in the built environments was in accordance with the values reported by previous studies (39), and significantly higher alpha-diversity was observed in road dust than in floor surfaces, suggesting higher species number and species evenness for road dust.

Microbial communities were significantly clustered with habitats, and the shared taxa for each habitat occupied a large proportion of microbial community exclusively in their respective habitat, indicating that microbial distribution patterns could be strongly driven by habitats. The disparities of environmental conditions in the investigated habitats, such as the availability of nutrients, humidity, air exchange rate, temperature fluctuations, and UV radiation contributed to the spatial heterogeneity of the microbiomes (40). Distinct seasonal distribution of microbial communities was also observed in malls. The significant increase in the number of shoppers in summer could be an important factor for the seasonal distribution of microbial communities. With the increased occupant number in summer, the exchange of microorganisms between humans and the environment would be more frequent, resulting in different microbial compositions. It also should be noted that samples in this study were collected in spring and summer owing to the climatic conditions of the target city, a subtropical maritime climate without typical winter weather. For those cities with four distinct seasons, analysis of samples collected from each season would provide a more comprehensive profile of the seasonal distribution of microbial communities. Previous studies of the transit microbiome suggested that the surface types were the major determinant of the variations in microbial composition, and human-surface interactions largely shaped the community composition (4, 14, 15, 41). We also observed a significant enrichment of the human skin-associated microbiome on shopping mall floors and escalator handrails, suggesting that human-environment interactions were another strong driver for community patterns of the mall microbiome. The low sensitivity and high error ratios of RF classifiers for differentiating malls indicated the classification confusion for distinguishing one sample as having come from a specific mall. The possible explanations were that the similar environmental conditions, operational modes, and human activities across malls exert a homogenizing effect on mall surface microbial communities, hindering the precise discrimination of specific shopping malls (18). The sample size in each shopping mall is another important factor affecting the accuracy of models for differentiating the individual malls. The small data set with many outliers, missing values, or skewed data would impact the score of each decision tree of the random forest classifier.

Periodic anthropogenic perturbations in built environments (e.g., regular cleaning, disinfection, hand contact, and walking) would create opportunities for microorganisms to adapt to or colonize the environment and for communities to reassemble (42). Microorganisms can establish a range of relationships that generate increased benefits for the community. Microbial interaction networks may elucidate more about the inner workings of a community and the processes governing the assembly of community. Our analysis revealed that both bacterial and fungal networks consisted mainly of positively synergistic interactions. Indoor environments (floors and escalators) harbored more significant pairwise relationships, whereas more complex, dense, and robust microbial interconnections were observed in outdoor environment communities (soils and road dusts). In the surface environment, most microorganisms might be concurrently transported to the habitats from the same source through cleaning materials or walking, thus resulting in many pairwise relationships. However, regular cleaning and disinfection are possibly an important modifying factor for regulating the interactions of the surface microbiome with decreased microbial biomass and viability on surfaces, hence causing reduced microbial complex interactions. Soil and road dust would provide more nutrients or relatively stable conditions, resulting in a profitable niche for microbial fitness and interactions (43, 44).

The built environment is considered a microbial wasteland, where microbes passively accumulate and the process of microbial colonization is intrinsically stochastic (3, 43). The combination of biotic factors, such as the interactions between newcomers and resident microorganisms, and abiotic factors, such as nutrient availability, may change the process of community assembly (40, 43). We determined the ecological processes (neutral versus selective) underpinning microbial community assembly in these built environment habitats. The composition of the overall microbial community in these built environments was consistent with neutral model predictions, suggesting that the stochastic balance between the loss and gain of microbes (such as stochastic growth, death, and immigration) shaped the variations in the microbial communities (44). The neutral model generally incorporates passive dispersal (for example, sampling individuals from a source pool of available species) to ecological drift (random births and deaths of individuals) as a neutral process (45). However, owing to the microbial flux between human skin and floor/escalator and/or seasonal fluctuation, community dispersal might be the more relevant neutral process for floors and escalators. For soil and road dust, it is difficult to infer the relative role of dispersal versus ecological drift due to a lack of temporal data. We also observed minimum values of the NCM parameters *R*^2^, *Nm*, and *m* in the soil fungal community. This could be attributed largely to plant genotype and development in greenbelt soils, which exerted strong effects on microbiome assembly (46). The NST index demonstrated that the importance of stochastic processes was not similar between bacterial and fungal communities in the same habitat. Bacterial community assemblies were more stochastic than fungi in soil and road dust. The main possible explanation is that fungal hyphae penetrate or become entangled with the soil or dust aggregates, and the dispersal of the fungal community can be both deterministic and stochastic (47, 48).

Shopping malls are the main environment where people share microbes, and many diseases associated with human activities may have their origins in malls (10). Previous studies have revealed that pathogens and AMR were frequently detected in built environments, e.g., nosocomial infectious pathogens in hospital (19) and VAG- or ARG-carried microbes in metro systems (14, 15, 31), and could spread to humans by close interactions between individuals and surface microbiota. Our observation showed that 0.82% to 8.7% of potential human pathogens were identified in the core microbiomes of the built environments. HT-qPCR assays also revealed that human pathogens were prevalent and that indoor environments harbored more abundant pathogens than outdoor samples, indicating that human exposure to these mall surfaces might represent a potential health risk. Skin-to-surface direct contact and/or shedding of biological particles possibly introduces and transmits the microbial contaminants indoor (20, 49–51); humidity, air temperature, and the occupant density and source of ventilation air can also influence the abundance and transmission of pathogenic microorganisms in indoor environments (2).

*Enterobacteriaceae* species are important pathogens in health care- and community-associated infections worldwide, and the emergence and spread of resistance among *Enterobacteriaceae* species are threatening antibiotic treatment efficacy. Genomic analysis showed that a high diversity of AMR genes (e.g., *CRP*, *oqxB*, *acrA*, the H-NS gene, and *baeR*) encoding the multidrug efflux pump for antibiotics, disinfectants, and detergents (e.g., triclosan, fluoroquinolones, and cephalosporin) was detected in *Enterobacteriaceae* isolates. Previous reports suggested that the high proportions of multidrug efflux genes were frequently found in microbe-controlled compartments, including hospital-associated surfaces, intensive care units, and cleanroom facilities (10), and the multidrug efflux systems were one of the most frequently reported resistance mechanisms in *Enterobacteriaceae* species (8). Increased confinement and cleaning (regular exposure to disinfectants and cleaning reagents) in indoor environments were reported to be associated with an overall high level of virulence and antimicrobial resistance of microbial populations (10). Although tigecycline-resistant *tet* variants were not detected, the gene *oqxB* encoding RND type efflux pumps associated with tigecycline susceptibility was detected in resistant strains. Thus, the prevalence of the efflux pumps possibly also explained the incompatibility between genotype and phenotype of tigecycline-resistant strains (50, 52). Plasmid was not assembled in the present study, but the flanking regions of ARGs [e.g., *APH(6)-Id* encoding beta-lactamase] carried by several potential pathogens contained transposon-related genes (e.g., Tn*3*, IS*26*, and *insF*), suggesting potential transposon-mediated horizontal transfer of ARGs between human pathogens and other environmental strains.

## MATERIALS AND METHODS

### Sample collection.

Surface samples from 20 shopping malls were collected in March (spring, average daytime temperature of 21.6°C and relative humidity of 72%) and July (summer, average daytime temperature of 35°C and relative humidity of 85%) of 2020 in Xiamen, China (see Table S1 in the supplemental material). Samples were not collected in autumn and winter. Since the target city, Xiamen, is a subtropical city near the Tropic of Cancer, without typical winter, the weather conditions are similar in spring and autumn. Air humidity in shopping malls was kept in the range of 50% to 60%, and the temperature was 22°C to 27°C (https://www.cma.gov.cn/). We noticed that the occupancy in malls dramatically increased in summer compared to spring owing to the mitigation of the COVID-19 pandemic after April 2020 in China (https://new.qq.com/rain/a/20210312A0572S00). For all malls, cleaning and sanitizing (commonly with sodium hypochlorite) were regularly performed each day before opening (7:00 a.m. to 9:00 a.m.). Discontinuous cleaning operations were also performed during daily business hours according to foot traffic. Sampling time was set to the preferred afternoon time (3:00 p.m. to 6:00 p.m.) of shoppers.

A sterile nylon-flocked swab with 1 mL of transport medium (liquid Amies elution swab 481C; Copan, Italy) was used for collecting surface samples according to a previous study by Afshinnekoo et al. (14). Briefly, two swabs from floor surfaces close to each entrance for one shopping mall were taken by swabbing an area of approximately 6 cm by 6 cm for 1 min. For the escalator surfaces, only the right handrails of both ascending and descending escalators were swabbed with a palm-size (~0.01-m^2^) surface area during one transfer. Swabs were immediately placed into collection tubes, with immersion in the transport medium. To detect background contamination caused by air biological materials during sample collection, a buffer-dampened empty swab was held in the air for 1 min in each mall as a negative control. Road dust was collected from the streets (approximately 50 to 100 m away from the malls) around 12 shopping malls by using 75% ethanol-sterilized brushes or vacuum cleaners. To achieve adequate dust, road dust samples from more than two sites on the same street were pooled, and the sampling area for each site depended on the amount of available dust. Approximately 50 g of greenbelt surface soil (~0 to 20 cm) in close proximity to the road dust sampling sites was also sampled using sterile scoops. A total of 474 samples were collected, including 222 floor swabs, 218 escalator swabs, 27 soil samples, and 27 road dust samples. The collected samples were immediately kept on ice and then transported to a −20°C freezer within 3 h.

### DNA extraction and amplicon sequencing.

Surface samples in transport medium were thawed at room temperature, and the entire nylon fiber flocked swab tips were cut by sterile scissors and sterilized with 75% ethanol before passage through a flame. Then, the medium and the swab tip for one sample were transferred into a collection tube in a FastDNA spin kit for soil DNA extraction (MP Biomedicals, USA). To obtain adequate DNA, DNA from two swabs from the same entrance/escalator was pooled in 70 μL of elution buffer. For road dust and soils, 0.5-g samples were used for DNA extraction. Thus, 274 DNA samples were used for microbial community analysis, including 111 DNA samples from floors, 109 from escalators, 27 from road dust, and 27 from soils (Table S1). DNA concentrations were measured by a Quant-iT double-stranded DNA (dsDNA) high-sensitivity assay kit (Invitrogen) and a Qubit 3.0 fluorometer (Invitrogen) according to the protocols provided. DNA extraction and amplification for negative controls were performed using the same protocol.

Amplicons for bacterial 16S rRNA gene were generated using the barcoded primers 515F and 806R (53), and the fungal internal transcribed spacer (ITS) was amplified with the barcoded primers ITS1F and ITS2R (39). A DNA library was constructed and amplicon sequencing was performed on an Illumina MiSeq PE250 sequencing instrument (Shanghai Majorbio, China). Further details for amplicon sequencing can be found in Text S1 in the supplemental material. The DNA concentrations of negative controls were below the detection limit of a Qubit 3.0 fluorometer (Invitrogen), and amplification of the 16S rRNA gene and ITS returned negative results.

10.1128/msystems.00576-22.10 TEXT S1 Detailed information for amplicon sequencing, HT-qPCR assays for human pathogens, identification of the *Enterobacteriaceae* isolates from malls, neutral community models, and random forest models. Download Text S1, DOCX file, 0.04 MB. Copyright © 2022 An et al. 2022 An et al. https://creativecommons.org/licenses/by/4.0/ This content is distributed under the terms of the Creative Commons Attribution 4.0 International license.

### HT-qPCR assays for human pathogens.

TaqMan-based HT-qPCR analysis for human pathogens was performed by using a WaferGen SmartChip real-time PCR system (TaKaRa, Japan) according to our previous study (54). HT-qPCR assays simultaneously quantify 68 marker genes for 33 human pathogens that are commonly associated with respiratory infections, intestinal illness, keratitis, and other diseases. Data analysis for HT-qPCR was performed according to the methods in Text S1 in the supplemental material.

### Isolation and antimicrobial susceptibility testing of *Enterobacteriaceae* species.

Coliforms were isolated from the floor and escalator transport samples by using CHROMagar ECC agar (France) in accordance with the manufacturer’s protocol. Taxonomic identification of the isolates was performed by sequencing the full-length 16S rRNA gene (Text S1). Antibiotic susceptibility to meropenem, cephalothin, tigecycline, and ertapenem (Oxoid, UK) was determined for the identified *Enterobacteriaceae* species using the disc diffusion method according to the European Committee on Antimicrobial Susceptibility Testing (EUCAST, version 10.0; https://www.eucast.org/) and the Clinical and Laboratory Standards Institute (CLSI 2015, M100-S25; https://clsi.org/). Reference strain Escherichia coli DH5α served as the quality control strain.

### Whole-genome sequencing of *Enterobacteriaceae* species.

*Enterobacteriaceae* isolate DNA was extracted using a Wizard genomic DNA purification kit (Promega, USA). Indexed DNA libraries were constructed with an insert size of 300 bp by using a NEBNext Ultra DNA library prep kit (New England Biolabs, USA), and sequencing was performed on an Illumina HiSeq × 10 system with a minimum of 100-fold coverage (Shanghai Majorbio, China).

### Bioinformatics.

**(i) Phylotype analysis for amplicons.** Raw sequencing data of 16S rRNA/ITS gene amplicons were demultiplexed by tag sequences using QIIME python scripts (split_libraries_fastq.py) (55); the DADA2 pipeline based on amplicon sequence variants (ASVs) was applied for microbial diversity analysis. Briefly, the core sample inference algorithm was used for filtering and trimming demultiplexed sequences (maximum number of N [maxN] = 0, maximum number of expected errors allowed in a read [maxEE] = 2, and DADA2 truncate the read at the first nucleotide with a quality score of 2 [truncQ = 2]) (56). Subsequently, error rates were checked, deduplication was performed, and forward/reverse reads were merged. Chimeras were removed from the merged sequences, and a feature table of ASVs was generated. Singletons, chloroplasts, and mitochondrial sequences were removed from the final data set. The sequence variants were assigned to taxonomic lineages against the SILVA reference database (version 132) for bacteria and the UNITE ITS database for fungi by using a naive Bayesian classifier method (57).

**(ii) Whole-genome sequencing analysis.** Sequencing adapters were removed, and quality filtering of the acquired reads was conducted using FastQC. Draft genomes were *de novo* assembled using SOAPdenovo2 (58) and visualized by CLC Genomics Workbench 8 (CLC Bio). The putative coding sequences (CDS) were predicted using Glimmer v3.02 (59). Protein sequences were converted from CDS and annotated using Diamond against the NR, Swiss-Prot, Pfam, EggNOG, GO, and KEGG databases (E value ≤ 1e−5). ARGs were identified by matching the sequences to the Comprehensive Antibiotic Resistance Database (CARD, version 1.1.3) (60) and the ResFinder database (61). VAGs were annotated by searching the assembled contigs against the virulence factor database VFDB (62). Integron_Finder was applied to identify the integron by detecting the promoters and *attI* sites with the use of INFERNAL and HMMER profiles (43–65). These genes were screened with a strict cutoff value of amino acid identity of ≥98% and a query coverage of ≥99%. Phylogenetic trees based on the alignments of full-length 16S rRNA gene sequences and SNPs in the core genomes were constructed using PhyML based on a maximum likelihood approach (bootstrap = 10,000) and displayed using iTOL (66).

### Statistical analysis.

Analysis for microbial alpha-diversity and beta-diversity was performed in the open-source R environment (v4.0.3) with the package vegan 2.2.0 (67). Three alpha-diversity indices, including Shannon, Simpson, and Chao 1 indexes, were calculated, and the beta-diversity distribution variation was evaluated using Bray-Curtis dissimilarity-based nonmetric multidimensional scaling (NMDS) or principal-coordinate analysis (PCoA). A permutational multivariate analysis of variance (PERMANOVA, Adonis test) was performed to assess the significance of dissimilarity in microbial beta-diversity distribution. To understand the interactions between microorganisms, correlation analysis of microbial taxa at the family level was conducted using a pairwise Pearson method, and ASV networks were visualized using Gephi. *P* values were adjusted for comparisons with the false discovery rate (FDR) algorithm to reduce the false-positive results (68). For comparative analysis between seasons/sample types, paired *t* tests and one-way ANOVA tests were performed using SPSS (IBM). All statistical tests were considered significant at a *P *of <0.05. To determine the potential sources of the indoor (floors and escalators) microbial communities, we downloaded the data sets of 16S rRNA gene amplicons from previous studies of the outdoor air microbiome (Xiamen, China) and the human skin (palm) microbiome (69, 70). The microbiota from road dust, greenbelt soils, outdoor air, and human palms were used as the sources, and microbial communities on floors and escalators were treated as the sinks. SourceTracker models were constructed using Bayesian SourceTracker in QIIME with default settings (http://qiime.org/tutorials/source_tracking.html) (71). We also assessed the stochasticity of community assembly using a neutral community model (NCM) and a normalized stochasticity ratio (NST) index. The relationships between the detection frequency of microbial taxa and their relative abundance across the wider metacommunity were predicted in the NCM models (72). Random forest (RF) supervised learning models were trained for illuminating the forensic potential of the microbiome and predicting shopping mall, season, and sample type classifications (73). More details about NCM and RF models can be found in Text S1.

Our study represents a comprehensive analysis of the mall microbiome and provides a better understanding of microbial distribution, assembly, pathogen distributions, and antimicrobial resistance burdens in shopping malls and their adjacent road dust and soils. It was demonstrated that these shopping mall-related habitats had distinct bacterial community compositions, and environmental factors (seasons and habitats) and human activities commonly affected the variations in microbial communities. The identification of microbial signatures indicated the predictive potential of microbial communities for shopping mall, season, and habitat discrimination. Stochastic processes largely contributed to the microbial community assembly. Additionally, more human pathogens were observed in indoor environments, carrying a high proportion of antimicrobial resistance-associated multidrug efflux genes and virulence genes. These data provide a background baseline for further study of the mall microbiome by integrating multidimensional factors from human population, time, environment, and geography. A dynamic surveillance of human pathogens and antimicrobial resistance in malls could also provide scientific data for public policymaking regarding environmentally mediated transmission of harmful microorganisms.

### Data availability.

The raw amplicon sequences were submitted to the Sequence Read Archive (SRA) under accession no. PRJNA707496. All full-length sequences of 16S rRNA genes and the genome assemblies of *Enterobacteriaceae* species were deposited under GenBank no. MZ461606–MZ461903 and SRA accession no. PRJNA749475, respectively.
