# Supplementary figures and images for: Dynamics of Microbial Community and Potential Microbial Pollutants in Shopping Malls
Source: mSystems. 2023 Jan 5;8(1):e00576-22. doi: 10.1128/msystems.00576-22 (PMC9948725; doi:10.1128/msystems.00576-22)

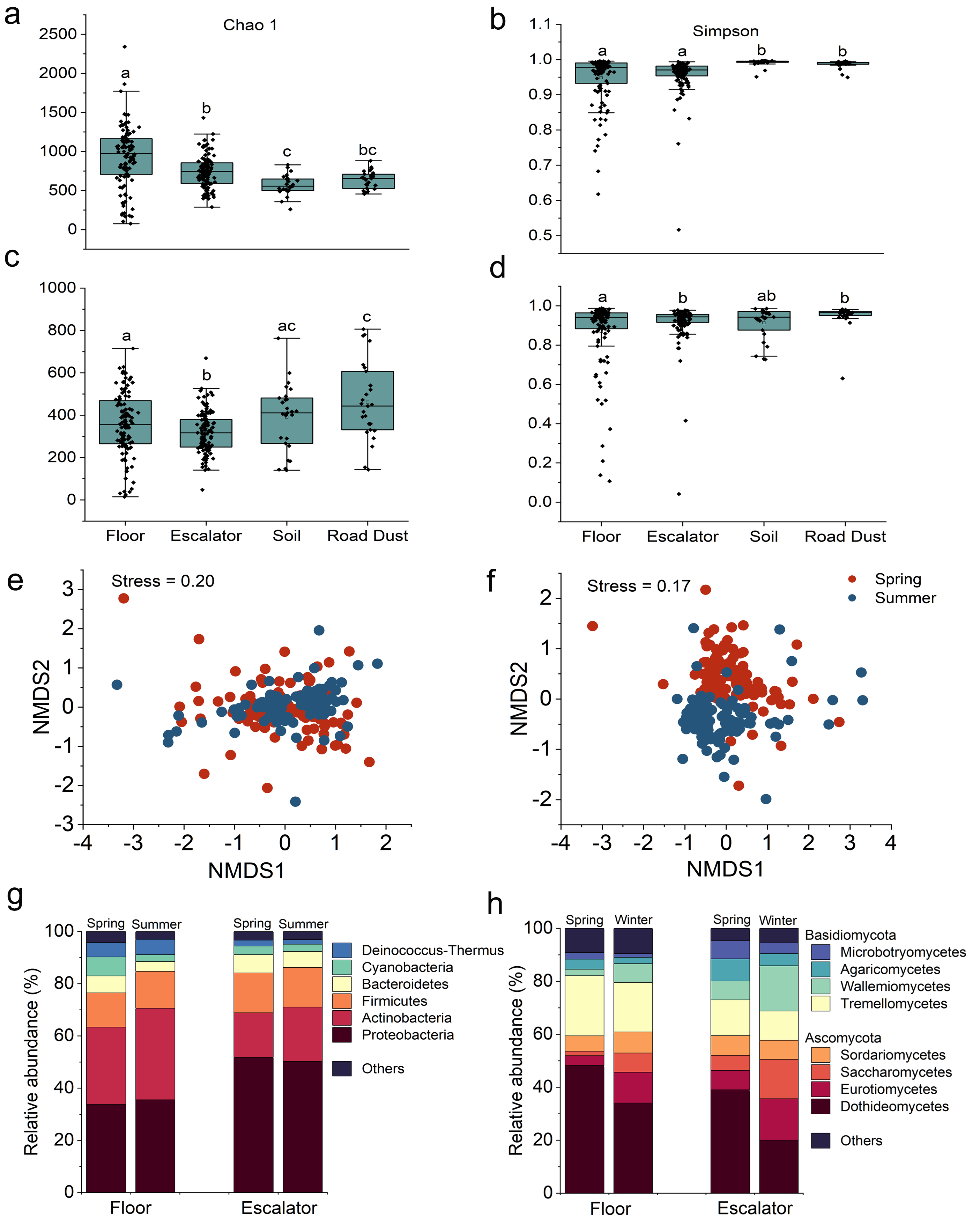

Supplement: FIG S1 [file msystems.00576-22-s0002.tif]

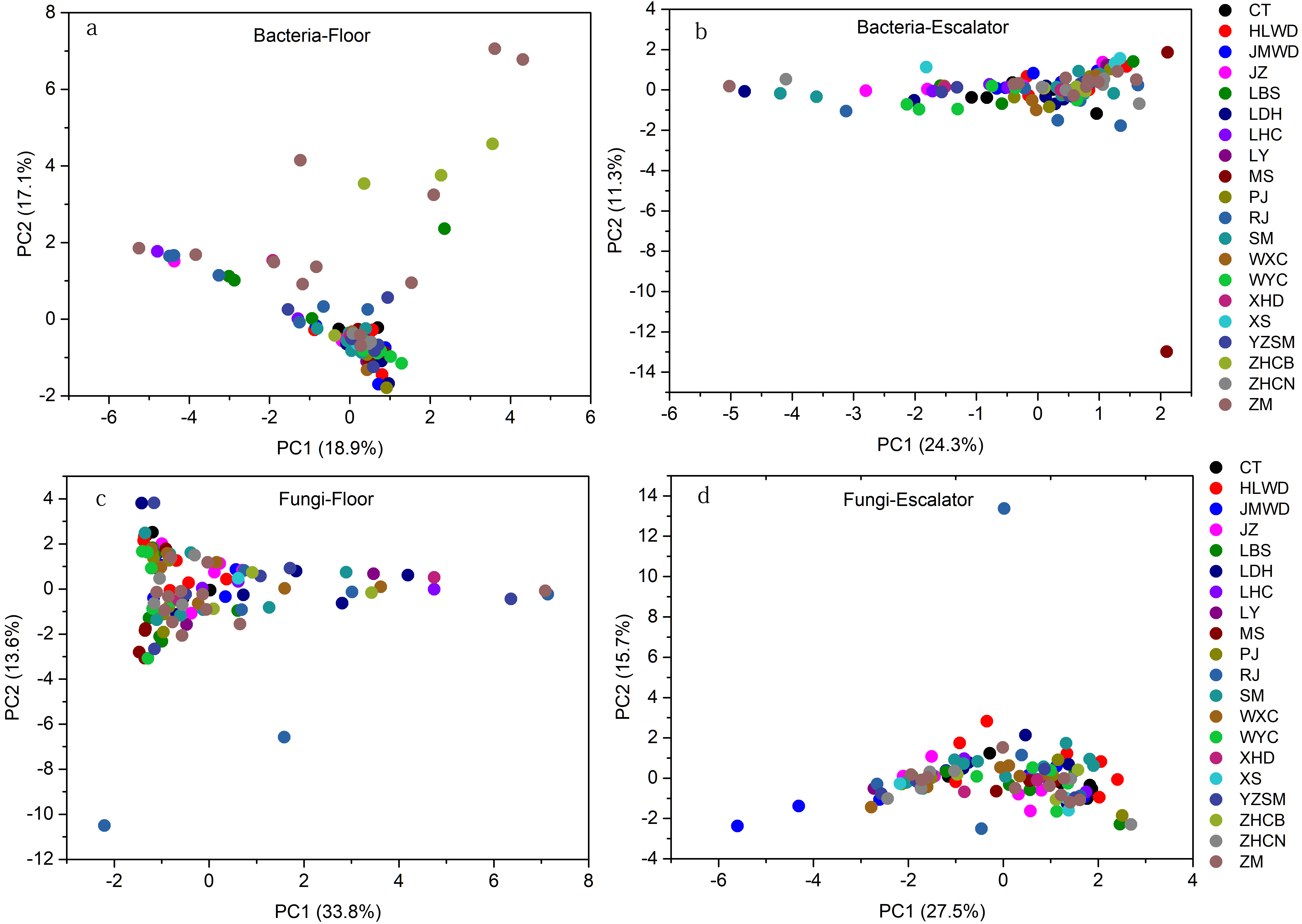

Supplement: FIG S2 [file msystems.00576-22-s0003.tif]

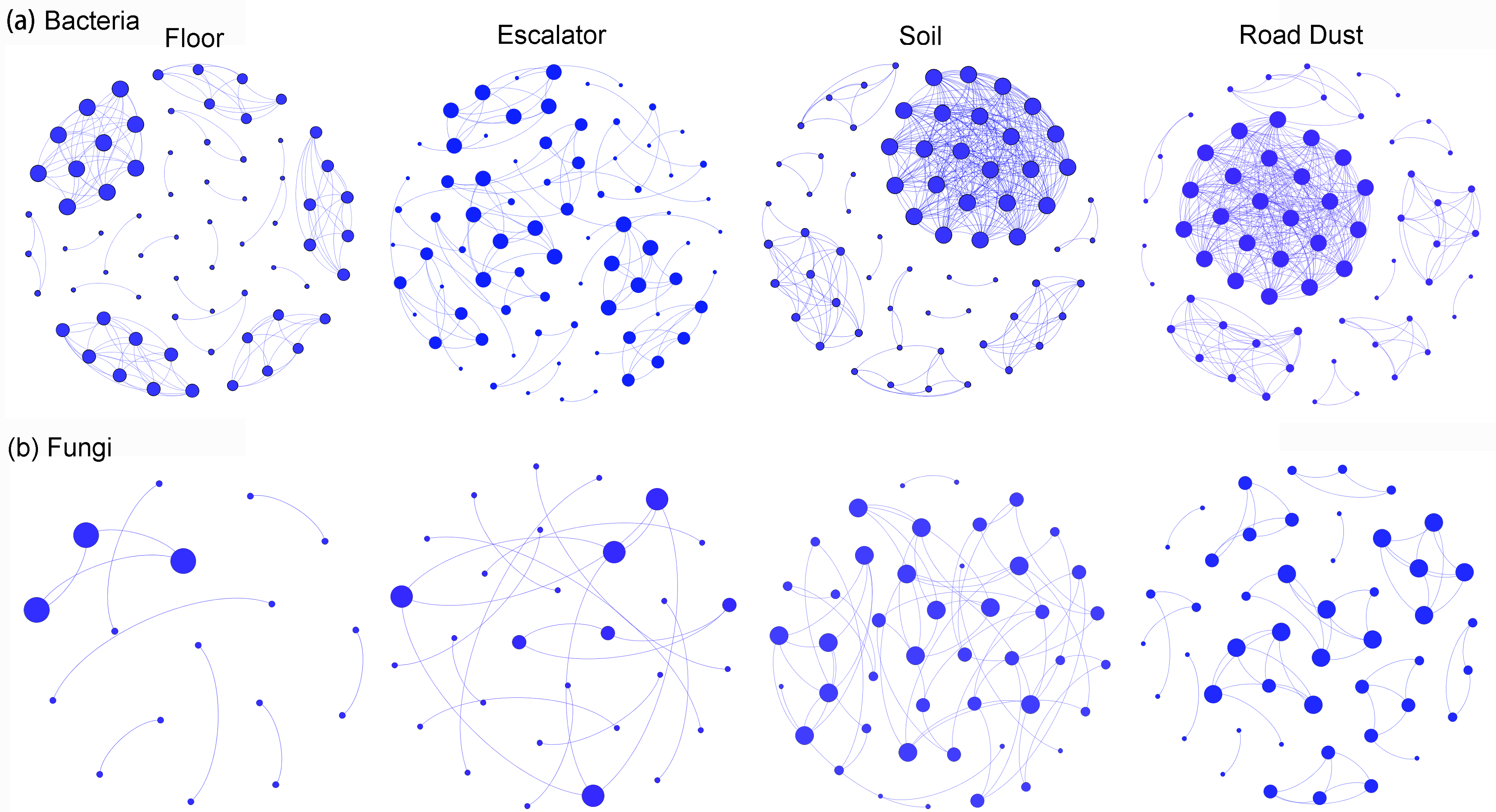

Supplement: FIG S3 [file msystems.00576-22-s0004.tif]

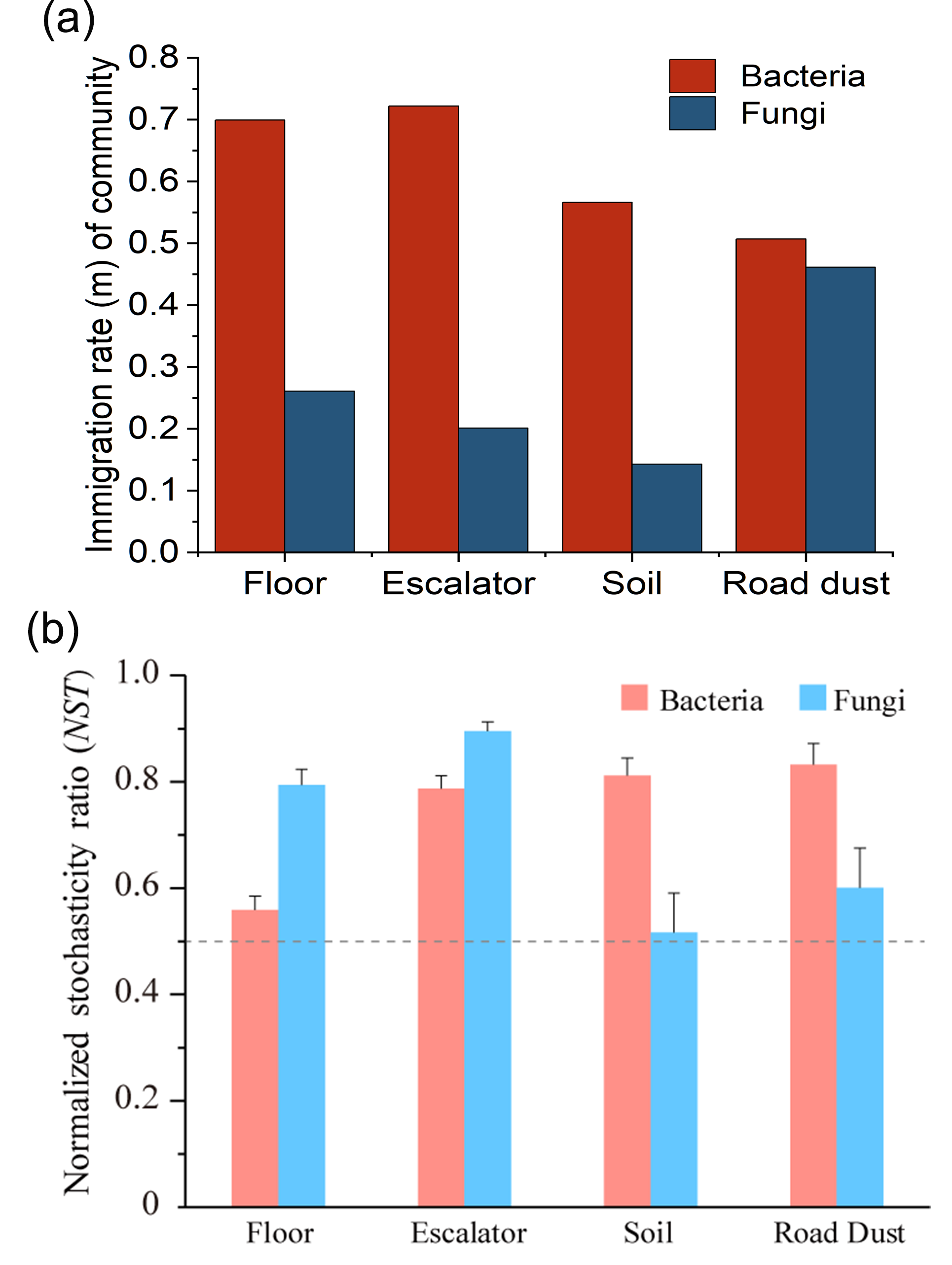

Supplement: FIG S4 [file msystems.00576-22-s0005.tif]

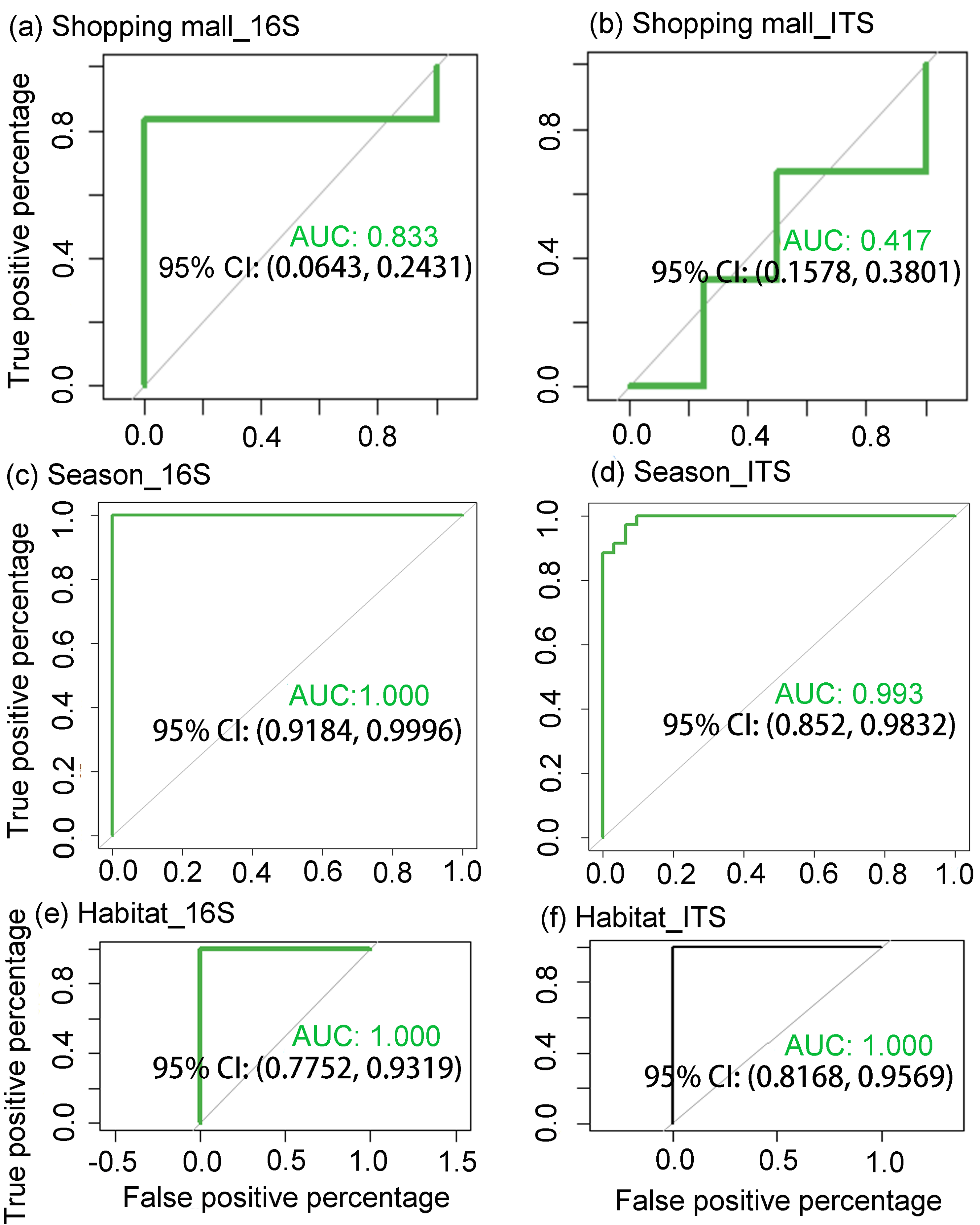

Supplement: FIG S5 [file msystems.00576-22-s0006.tif]

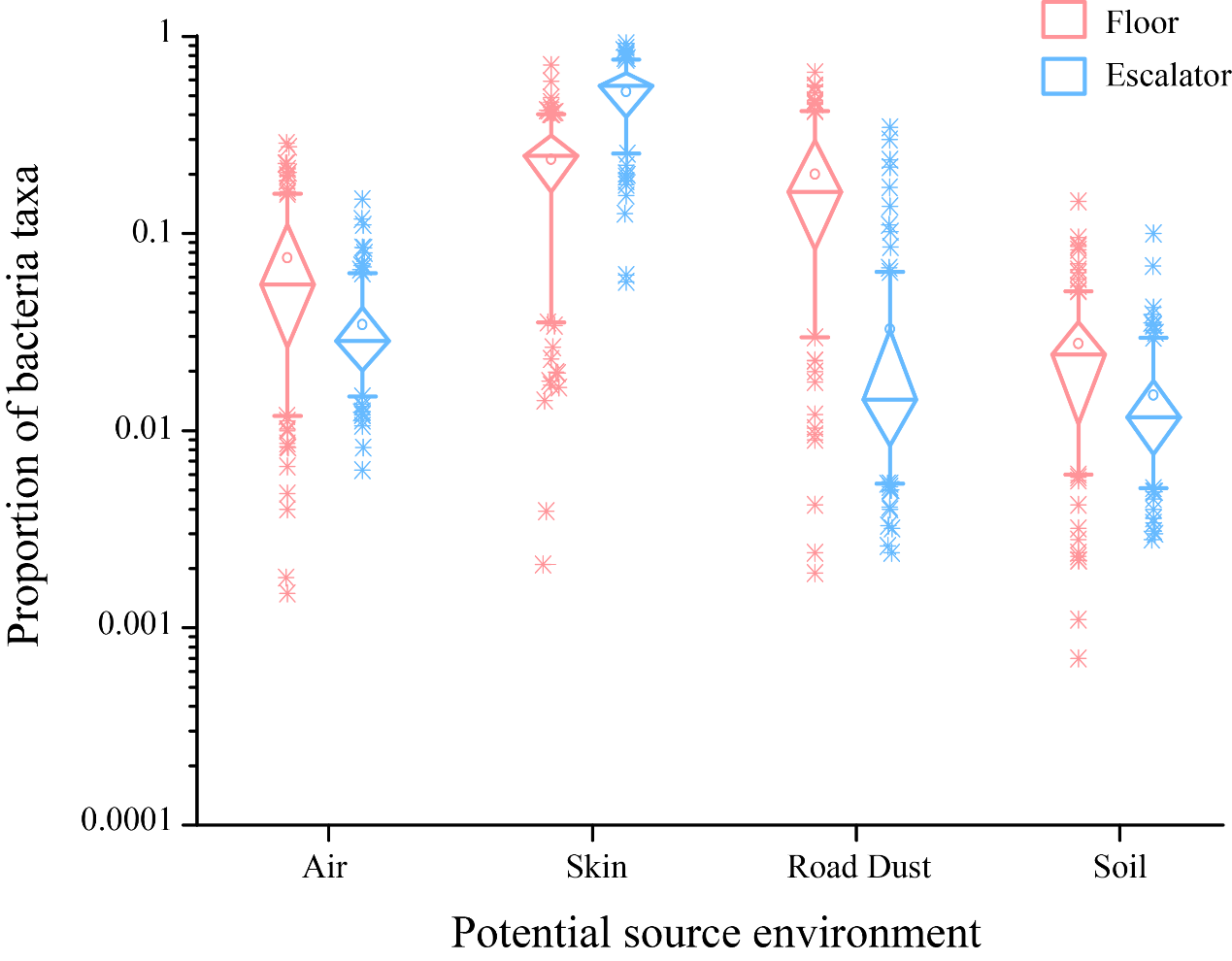

Supplement: FIG S6 [file msystems.00576-22-s0007.tif]
