# Supplementary material for: Dynamics of Microbial Community and Potential Microbial Pollutants in Shopping Malls
Source: mSystems. 2023 Jan 5;8(1):e00576-22. doi: 10.1128/msystems.00576-22 (PMC9948725; doi:10.1128/msystems.00576-22)
Supplement: TABLE S1 [file msystems.00576-22-s0008.docx]

**Table S1 Summary for the collected samples.**

| **DNA Sample Names*** | **Habitats** | **Sampling Time** | **Shopping Mall Name** | **Number of Entrances** | **Number of Escalators** |
| --- | --- | --- | --- | --- | --- |
| LBS_F1_1 | Floor | March, 2020 | LBS | 3 |  |
| LBS_F1_2 | Floor | March, 2020 |  |  |  |
| LBS_F1_3 | Floor | March, 2020 |  |  |  |
| YZSM_F1_1 | Floor | March, 2020 | YZSM | 3 |  |
| YZSM_F1_2 | Floor | March, 2020 |  |  |  |
| YZSM_F1_3 | Floor | March, 2020 |  |  |  |
| ZM_F1_1 | Floor | March, 2020 | ZM | 6 |  |
| ZM_F1_2 | Floor | March, 2020 |  |  |  |
| ZM_F1_3 | Floor | March, 2020 |  |  |  |
| ZM_F1_4 | Floor | March, 2020 |  |  |  |
| ZM_F1_5 | Floor | March, 2020 |  |  |  |
| ZM_F1_6 | Floor | March, 2020 |  |  |  |
| WXC_F1_1 | Floor | March, 2020 | WXC | 3 |  |
| WXC_F1_2 | Floor | March, 2020 |  |  |  |
| WXC_F1_3 | Floor | March, 2020 |  |  |  |
| PJ_F1_1 | Floor | March, 2020 | PJ | 3 |  |
| PJ_F1_2 | Floor | March, 2020 |  |  |  |
| PJ_F1_3 | Floor | March, 2020 |  |  |  |
| HLWD_F1_1 | Floor | March, 2020 | HLWD | 4 |  |
| HLWD_F1_2 | Floor | March, 2020 |  |  |  |
| HLWD_F1_3 | Floor | March, 2020 |  |  |  |
| HLWD_F1_4 | Floor | March, 2020 |  |  |  |
| CT_F1_1 | Floor | March, 2020 | CT | 3 |  |
| CT_F1_2 | Floor | March, 2020 |  |  |  |
| CT_F1_3 | Floor | March, 2020 |  |  |  |
| ZHCN_F1_1 | Floor | March, 2020 | ZHCN | 2 |  |
| ZHCN_F1_2 | Floor | March, 2020 |  |  |  |
| ZHCB_F1_1 | Floor | March, 2020 | ZHCB | 2 |  |
| ZHCB_F1_2 | Floor | March, 2020 |  |  |  |
| LHC_F1_1 | Floor | March, 2020 | LHC | 2 |  |
| LHC_F1_2 | Floor | March, 2020 |  |  |  |
| LY_F1 | Floor | March, 2020 | LY | 1 |  |
| WYC_F1_1 | Floor | March, 2020 | WYC | 3 |  |
| WYC_F1_2 | Floor | March, 2020 |  |  |  |
| WYC_F1_3 | Floor | March, 2020 |  |  |  |
| LDH_F1_1 | Floor | March, 2020 | LDH | 3 |  |
| LDH_F1_2 | Floor | March, 2020 |  |  |  |
| LDH_F1_3 | Floor | March, 2020 |  |  |  |
| JZ_F1_1 | Floor | March, 2020 | JZ | 3 |  |
| JZ_F1_2 | Floor | March, 2020 |  |  |  |
| JZ_F1_3 | Floor | March, 2020 |  |  |  |
| RJ_F1_1 | Floor | March, 2020 | RJ | 3 |  |
| RJ_F1_2 | Floor | March, 2020 |  |  |  |
| RJ_F1_3 | Floor | March, 2020 |  |  |  |
| SM_F1_1 | Floor | March, 2020 | SM | 4 |  |
| SM_F1_2 | Floor | March, 2020 |  |  |  |
| SM_F1_3 | Floor | March, 2020 |  |  |  |
| SM_F1_4 | Floor | March, 2020 |  |  |  |
| JMWD_F1_1 | Floor | March, 2020 | JMWD | 3 |  |
| JMWD_F1_2 | Floor | March, 2020 |  |  |  |
| JMWD_F1_3 | Floor | March, 2020 |  |  |  |
| MS_F1_1 | Floor | March, 2020 | MS | 3 |  |
| MS_F1_2 | Floor | March, 2020 |  |  |  |
| MS_F1_3 | Floor | March, 2020 |  |  |  |
| XHD_F1 | Floor | March, 2020 | XHD | 1 |  |
| LBS_E1_1 | Escalator | March, 2020 | LBS |  | 3 |
| LBS_E1_2 | Escalator | March, 2020 |  |  |  |
| LBS_E1_3 | Escalator | March, 2020 |  |  |  |
| YZSM_E1_1 | Escalator | March, 2020 | YZSM |  | 2 |
| YZSM_E1_2 | Escalator | March, 2020 |  |  |  |
| ZM_E1_1 | Escalator | March, 2020 | ZM |  | 6 |
| ZM_E1_2 | Escalator | March, 2020 |  |  |  |
| ZM_E1_3 | Escalator | March, 2020 |  |  |  |
| ZM_E1_4 | Escalator | March, 2020 |  |  |  |
| ZM_E1_5 | Escalator | March, 2020 |  |  |  |
| ZM_E1_6 | Escalator | March, 2020 |  |  |  |
| WXC_E1_1 | Escalator | March, 2020 | WXC |  | 3 |
| WXC_E1_2 | Escalator | March, 2020 |  |  |  |
| WXC_E1_3 | Escalator | March, 2020 |  |  |  |
| PJ_E1_1 | Escalator | March, 2020 | PJ |  | 3 |
| PJ_E1_2 | Escalator | March, 2020 |  |  |  |
| PJ_E1_3 | Escalator | March, 2020 |  |  |  |
| HLWD_E1_1 | Escalator | March, 2020 | HLWD |  | 3 |
| HLWD_E1_2 | Escalator | March, 2020 |  |  |  |
| HLWD_E1_3 | Escalator | March, 2020 |  |  |  |
| CT_E1_1 | Escalator | March, 2020 | CT |  | 3 |
| CT_E1_2 | Escalator | March, 2020 |  |  |  |
| CT_E1_3 | Escalator | March, 2020 |  |  |  |
| ZHCN_E1_1 | Escalator | March, 2020 | ZHCN |  | 3 |
| ZHCN_E1_2 | Escalator | March, 2020 |  |  |  |
| ZHCN_E1_3 | Escalator | March, 2020 |  |  |  |
| ZHCB_E1 | Escalator | March, 2020 | ZHCB |  | 1 |
| LHC_E1_1 | Escalator | March, 2020 | LHC |  | 2 |
| LHC_E1_2 | Escalator | March, 2020 |  |  |  |
| LY_E1 | Escalator | March, 2020 | LY |  | 1 |
| XS_E1 | Escalator | March, 2020 | XS |  | 1 |
| WYC_E1_1 | Escalator | March, 2020 | WYC |  | 3 |
| WYC_E1_2 | Escalator | March, 2020 |  |  |  |
| WYC_E1_3 | Escalator | March, 2020 |  |  |  |
| LDH_E1_1 | Escalator | March, 2020 | LDH |  | 3 |
| LDH_E1_2 | Escalator | March, 2020 |  |  |  |
| LDH_E1_3 | Escalator | March, 2020 |  |  |  |
| JZ_E1_1 | Escalator | March, 2020 | JZ |  | 3 |
| JZ_E1_2 | Escalator | March, 2020 |  |  |  |
| JZ_E1_3 | Escalator | March, 2020 |  |  |  |
| RJ_E1_1 | Escalator | March, 2020 | RJ |  | 3 |
| RJ_E1_2 | Escalator | March, 2020 |  |  |  |
| RJ_E1_3 | Escalator | March, 2020 |  |  |  |
| SM_E1_1 | Escalator | March, 2020 | SM |  | 5 |
| SM_E1_2 | Escalator | March, 2020 |  |  |  |
| SM_E1_3 | Escalator | March, 2020 |  |  |  |
| SM_E1_4 | Escalator | March, 2020 |  |  |  |
| SM_E1_5 | Escalator | March, 2020 |  |  |  |
| JMWD_E1_1 | Escalator | March, 2020 | JMWD |  | 3 |
| JMWD_E1_2 | Escalator | March, 2020 |  |  |  |
| JMWD_E1_3 | Escalator | March, 2020 |  |  |  |
| XHD_E1 | Escalator | March, 2020 | XHD |  | 1 |
| LBS_F2_1 | Floor | June, 2020 | LBS | 3 |  |
| LBS_F2_2 | Floor | June, 2020 |  |  |  |
| LBS_F2_3 | Floor | June, 2020 |  |  |  |
| YZSM_F2_1 | Floor | June, 2020 | YZSM | 3 |  |
| YZSM_F2_2 | Floor | June, 2020 |  |  |  |
| YZSM_F2_3 | Floor | June, 2020 |  |  |  |
| ZM_F2_1 | Floor | June, 2020 | ZM | 6 |  |
| ZM_F2_2 | Floor | June, 2020 |  |  |  |
| ZM_F2_3 | Floor | June, 2020 |  |  |  |
| ZM_F2_4 | Floor | June, 2020 |  |  |  |
| ZM_F2_5 | Floor | June, 2020 |  |  |  |
| ZM_F2_6 | Floor | June, 2020 |  |  |  |
| WXC_F2_1 | Floor | June, 2020 | WXC | 4 |  |
| WXC_F2_2 | Floor | June, 2020 |  |  |  |
| WXC_F2_3 | Floor | June, 2020 |  |  |  |
| WXC_F2_4 | Floor | June, 2020 |  |  |  |
| PJ_F2_1 | Floor | June, 2020 | PJ | 4 |  |
| PJ_F2_2 | Floor | June, 2020 |  |  |  |
| PJ_F2_3 | Floor | June, 2020 |  |  |  |
| PJ_F2_4 | Floor | June, 2020 |  |  |  |
| HLWD_F2_1 | Floor | June, 2020 | HLWD | 4 |  |
| HLWD_F2_2 | Floor | June, 2020 |  |  |  |
| HLWD_F2_3 | Floor | June, 2020 |  |  |  |
| HLWD_F2_4 | Floor | June, 2020 |  |  |  |
| CT_F2_1 | Floor | June, 2020 | CT | 3 |  |
| CT_F2_2 | Floor | June, 2020 |  |  |  |
| CT_F2_3 | Floor | June, 2020 |  |  |  |
| ZHCN_F2_1 | Floor | June, 2020 | ZHCN | 1 |  |
| ZHCB_F2_1 | Floor | June, 2020 | ZHCB | 2 |  |
| ZHCB_F2_2 | Floor | June, 2020 |  |  |  |
| LHC_F2 | Floor | June, 2020 | LHC | 1 |  |
| LY_F2 | Floor | June, 2020 | LY | 1 |  |
| XS_F2 | Floor | June, 2020 | XS | 1 |  |
| WYC_F2_1 | Floor | June, 2020 | WYC | 3 |  |
| WYC_F2_2 | Floor | June, 2020 |  |  |  |
| WYC_F2_3 | Floor | June, 2020 |  |  |  |
| LDH_F2_1 | Floor | June, 2020 | LDH | 3 |  |
| LDH_F2_2 | Floor | June, 2020 |  |  |  |
| LDH_F2_3 | Floor | June, 2020 |  |  |  |
| JZ_F2_1 | Floor | June, 2020 | JZ | 3 |  |
| JZ_F2_2 | Floor | June, 2020 |  |  |  |
| JZ_F2_3 | Floor | June, 2020 |  |  |  |
| RJ_F2_1 | Floor | June, 2020 | RJ | 3 |  |
| RJ_F2_2 | Floor | June, 2020 |  |  |  |
| RJ_F2_3 | Floor | June, 2020 |  |  |  |
| SM_F2_1 | Floor | June, 2020 | SM | 4 |  |
| SM_F2_2 | Floor | June, 2020 |  |  |  |
| SM_F2_3 | Floor | June, 2020 |  |  |  |
| SM_F2_4 | Floor | June, 2020 |  |  |  |
| JMWD_F2_1 | Floor | June, 2020 | JMWD | 3 |  |
| JMWD_F2_2 | Floor | June, 2020 |  |  |  |
| JMWD_F2_3 | Floor | June, 2020 |  |  |  |
| MS_F2_1 | Floor | June, 2020 | MS | 3 |  |
| MS_F2_2 | Floor | June, 2020 |  |  |  |
| MS_F2_3 | Floor | June, 2020 |  |  |  |
| XHD_F2 | Floor | June, 2020 | XHD | 1 |  |
| LBS_E2_1 | Escalator | June, 2020 | LBS |  | 3 |
| LBS_E2_2 | Escalator | June, 2020 |  |  |  |
| LBS_E2_3 | Escalator | June, 2020 |  |  |  |
| YZSM_E2_1 | Escalator | June, 2020 | YZSM |  | 2 |
| YZSM_E2_2 | Escalator | June, 2020 |  |  |  |
| ZM_E2_1 | Escalator | June, 2020 | ZM |  | 4 |
| ZM_E2_2 | Escalator | June, 2020 |  |  |  |
| ZM_E2_3 | Escalator | June, 2020 |  |  |  |
| ZM_E2_4 | Escalator | June, 2020 |  |  |  |
| WXC_E2_1 | Escalator | June, 2020 | WXC |  | 3 |
| WXC_E2_2 | Escalator | June, 2020 |  |  |  |
| WXC_E2_3 | Escalator | June, 2020 |  |  |  |
| PJ_E2_1 | Escalator | June, 2020 | PJ |  | 3 |
| PJ_E2_2 | Escalator | June, 2020 |  |  |  |
| PJ_E2_3 | Escalator | June, 2020 |  |  |  |
| HLWD_E2_1 | Escalator | June, 2020 | HLWD |  | 4 |
| HLWD_E2_2 | Escalator | June, 2020 |  |  |  |
| HLWD_E2_3 | Escalator | June, 2020 |  |  |  |
| HLWD_E2_4 | Escalator | June, 2020 |  |  |  |
| CT_E2_1 | Escalator | June, 2020 | CT |  | 3 |
| CT_E2_2 | Escalator | June, 2020 |  |  |  |
| CT_E2_3 | Escalator | June, 2020 |  |  |  |
| ZHCN_E2_1 | Escalator | June, 2020 | ZHCN |  | 3 |
| ZHCN_E2_2 | Escalator | June, 2020 |  |  |  |
| ZHCN_E2_3 | Escalator | June, 2020 |  |  |  |
| ZHCB_E2_1 | Escalator | June, 2020 | ZHCB |  | 2 |
| ZHCB_E2_2 | Escalator | June, 2020 |  |  |  |
| LHC_E2 | Escalator | June, 2020 | LHC |  | 1 |
| LY_E2 | Escalator | June, 2020 | LY |  | 1 |
| XS_E2_1 | Escalator | June, 2020 | XS |  | 2 |
| XS_E2_2 | Escalator | June, 2020 |  |  |  |
| WYC_E2_1 | Escalator | June, 2020 | WYC |  | 3 |
| WYC_E2_2 | Escalator | June, 2020 |  |  |  |
| WYC_E2_3 | Escalator | June, 2020 |  |  |  |
| LDH_E2_1 | Escalator | June, 2020 | LDH |  | 3 |
| LDH_E2_2 | Escalator | June, 2020 |  |  |  |
| LDH_E2_3 | Escalator | June, 2020 |  |  |  |
| JZ_E2_1 | Escalator | June, 2020 | JZ |  | 3 |
| JZ_E2_2 | Escalator | June, 2020 |  |  |  |
| JZ_E2_3 | Escalator | June, 2020 |  |  |  |
| RJ_E2_1 | Escalator | June, 2020 | RJ |  | 3 |
| RJ_E2_2 | Escalator | June, 2020 |  |  |  |
| RJ_E2_3 | Escalator | June, 2020 |  |  |  |
| SM_E2_1 | Escalator | June, 2020 | SM |  | 5 |
| SM_E2_2 | Escalator | June, 2020 |  |  |  |
| SM_E2_3 | Escalator | June, 2020 |  |  |  |
| SM_E2_4 | Escalator | June, 2020 |  |  |  |
| SM_E2_5 | Escalator | June, 2020 |  |  |  |
| JMWD_E2_1 | Escalator | June, 2020 | JMWD |  | 5 |
| JMWD_E2_2 | Escalator | June, 2020 |  |  |  |
| JMWD_E2_3 | Escalator | June, 2020 |  |  |  |
| JMWD_E2_4 | Escalator | June, 2020 |  |  |  |
| JMWD_E2_5 | Escalator | June, 2020 |  |  |  |
| MS_E2_1 | Escalator | June, 2020 | MS |  | 3 |
| MS_E2_2 | Escalator | June, 2020 |  |  |  |
| MS_E2_3 | Escalator | June, 2020 |  |  |  |
| XHD_E2 | Escalator | June, 2020 | XHD |  | 1 |
| ZHC_S | Soil | November, 2019 | ZHC |  |  |
| HLWD_S_1 | Soil | November, 2019 | HLWD |  |  |
| HLWD_S_2 | Soil | November, 2019 |  |  |  |
| HLWD_S_3 | Soil | November, 2019 |  |  |  |
| HLWD_S_4 | Soil | November, 2019 |  |  |  |
| ZM_S_1 | Soil | November, 2019 | ZM |  |  |
| ZM_S_2 | Soil | November, 2019 |  |  |  |
| JMWD_S_1 | Soil | November, 2019 | JMWD |  |  |
| JMWD_S_2 | Soil | November, 2019 |  |  |  |
| LBS_S_1 | Soil | November, 2019 | LBS |  |  |
| LBS_S_2 | Soil | November, 2019 |  |  |  |
| LBS_S_3 | Soil | November, 2019 |  |  |  |
| WXC_S_1 | Soil | November, 2019 | WXC |  |  |
| WXC_S_2 | Soil | November, 2019 |  |  |  |
| WXC_S_3 | Soil | November, 2019 |  |  |  |
| WYC_S_1 | Soil | November, 2019 | WYC |  |  |
| WYC_S_2 | Soil | November, 2019 |  |  |  |
| LDH_S_1 | Soil | November, 2019 | LDH |  |  |
| LDH_S_2 | Soil | November, 2019 |  |  |  |
| LDH_S_3 | Soil | November, 2019 |  |  |  |
| SM_S_1 | Soil | November, 2019 | SM |  |  |
| SM_S_2 | Soil | November, 2019 |  |  |  |
| RJ_S | Soil | November, 2019 | RJ |  |  |
| JZ_S_1 | Soil | November, 2019 | JZ |  |  |
| JZ_S_2 | Soil | November, 2019 |  |  |  |
| JZ_S_3 | Soil | November, 2019 |  |  |  |
| CT_S | Soil | November, 2019 | CT |  |  |
| ZHC_D | Road Dust | November, 2019 | ZHC |  |  |
| HLWD_D_1 | Road Dust | November, 2019 | HLWD |  |  |
| HLWD_D_2 | Road Dust | November, 2019 |  |  |  |
| HLWD_D_3 | Road Dust | November, 2019 |  |  |  |
| HLWD_D_4 | Road Dust | November, 2019 |  |  |  |
| ZM_D_1 | Road Dust | November, 2019 | ZM |  |  |
| ZM_D_2 | Road Dust | November, 2019 |  |  |  |
| JMWD_D_1 | Road Dust | November, 2019 | JMWD |  |  |
| JMWD_D_2 | Road Dust | November, 2019 |  |  |  |
| LBS_D_1 | Road Dust | November, 2019 | LBS |  |  |
| LBS_D_2 | Road Dust | November, 2019 |  |  |  |
| LBS_D_2 | Road Dust | November, 2019 |  |  |  |
| WXC_D_1 | Road Dust | November, 2019 | WXC |  |  |
| WXC_D_2 | Road Dust | November, 2019 |  |  |  |
| WXC_D_3 | Road Dust | November, 2019 |  |  |  |
| WYC_D_1 | Road Dust | November, 2019 | WYC |  |  |
| WYC_D_2 | Road Dust | November, 2019 |  |  |  |
| LDH_D_1 | Road Dust | November, 2019 | LDH |  |  |
| LDH_D_2 | Road Dust | November, 2019 |  |  |  |
| LDH_D_3 | Road Dust | November, 2019 |  |  |  |
| SM_D_1 | Road Dust | November, 2019 | SM |  |  |
| SM_D_2 | Road Dust | November, 2019 |  |  |  |
| RJ_D | Road Dust | November, 2019 | RJ |  |  |
| JZ_D_1 | Road Dust | November, 2019 | JZ |  |  |
| JZ_D_2 | Road Dust | November, 2019 |  |  |  |
| JZ_D_3 | Road Dust | November, 2019 |  |  |  |
| CT_D | Road Dust | November, 2019 | CT |  |  |

*indicates the DNA sample names (e.g., XX_AN_B) containing mall names (XX, abbreviation of mall names), habitat types [A, floor (F)/escalator (E)/soil (S)/dust (D)], sampling time [N, 1 (March, 2020)/2 (June, 2020)] and replicates (B, which replicate). For example, the DNA sample name LBS_F1_1 represents the DNA from the replicate 1 of floor surfaces of Luobinsen Mall collected in March, 2020.
