# Supplementary material for: Dynamics of Microbial Community and Potential Microbial Pollutants in Shopping Malls
Source: mSystems. 2023 Jan 5;8(1):e00576-22. doi: 10.1128/msystems.00576-22 (PMC9948725; doi:10.1128/msystems.00576-22)
Supplement: TABLE S2 [file msystems.00576-22-s0009.docx]

**Table S2 Topological features of co-occurrence networks for microbial communities.**

| Bacterial network | Dust-bacteria | Escalator-bacteria | Floor-bacteria | Soil-bacteria | Dust-fungi | Escalator-fungi | Floor-fungi | Soil-fungi |  |
| --- | --- | --- | --- | --- | --- | --- | --- | --- | --- |
| Number of significant correlations (*P* < 0.05) | 6435 | 9837 | 12621 | 4374 | 2269 | 2644 | 5961 | 4479 |  |
| Number of significantly negative correlations | 137 | 24 | 23 | 12 | 42 | 108 | 52 | 42 |  |
| Number of significantly positive correlations | 6298 | 9813 | 12598 | 4362 | 4438 | 4206 | 5909 | 4437 |  |
| Clustering coefficient | 0.88 | 0.62 | 0.66 | 0.87 | 0.63 | 0.24 | 0.18 | 0.79 |  |
| Number of nodes | 67 | 78 | 70 | 68 | 48 | 29 | 17 | 45 |  |
| Network heterogeneity | 0.85 | 0.59 | 0.71 | 0.86 | 0.45 | 0.52 | 0.32 | 0.40 |  |
| Connected components | 12 | 25 | 20 | 12 | 13 | 13 | 8 | 13 |  |
| Average number of neighbors | 10.93 | 2.74 | 4.14 | 10.77 | 2.54 | 1.38 | 1.18 | 2.71 |  |
