# Supplementary material for: Dynamics of Microbial Community and Potential Microbial Pollutants in Shopping Malls
Source: mSystems. 2023 Jan 5;8(1):e00576-22. doi: 10.1128/msystems.00576-22 (PMC9948725; doi:10.1128/msystems.00576-22)
Supplement: TABLE S3 [file msystems.00576-22-s0010.docx]

**Table S3** **Summary of predictive accuracy of random forest supervised learning models for habitat, season and shopping mall classification.**

| Class | Microbial samples | Sensitivity | Specificity | Positive Predictive Value | Negative Predictive Value | Balanced Accuracy |
| --- | --- | --- | --- | --- | --- | --- |
| Habitat classification | Floor-Bacteria | 0.9714 | 1 | 1 | 0.9796 | 0.9857 |
|  | Escalator-Bacteria | 1 | 0.9821 | 0.9643 | 1 | 0.9911 |
|  | Soil-Bacteria | 1 | 0.8611 | 0.5238 | 1 | 0.9306 |
|  | Dust-Bacteria | 0 | 1 | NA | 0.8795 | 0.5 |
|  | Floor-Fungi | 0.8788 | 0.9592 | 0.9355 | 0.9216 | 0.919 |
|  | Escalator-Fungi | 0.9394 | 0.9184 | 0.8857 | 0.9574 | 0.9289 |
|  | Soil-Fungi | 0.8 | 1 | 1 | 0.9729 | 0.9 |
|  | Dust-Fungi | 1 | 0.9737 | 0.75 | 1 | 0.9868 |
| Season classification | Season_Bacteria | 0.9688 | 1 | 1 | 0.9714 | 0.9844 |
|  | Season_Fungi | 0.9677 | 0.9143 | 0.9091 | 0.9697 | 0.941 |
| Shopping mall classification | CT_Bacteria | 0 | 0.88889 | 0 | 0.94915 | 0.44444 |
|  | HLWD_Bacteria | 0 | 0.91667 | 0 | 0.90164 | 0.45833 |
|  | JMWD_Bacteria | 0 | 1 | NA | 0.8939 | 0.5 |
|  | JZ_Bacteria | 0 | 0.9688 | 0 | 0.9688 | 0.4844 |
|  | LBS_Bacteria | 0.25 | 0.98387 | 0.5 | 0.95312 | 0.61694 |
|  | LDH_Bacteria | 0 | 0.98333 | 0 | 0.90769 | 0.49167 |
|  | LHC_Bacteria | 0 | 1 | NA | 0.9697 | 0.5 |
|  | LY_Bacteria | NA | 1 | NA | NA | NA |
|  | MS_Bacteria | 0 | 1 | NA | 0.95455 | 0.5 |
|  | PJ_Bacteria | 0.5 | 0.96875 | 0.33333 | 0.98413 | 0.73438 |
|  | RJ_Bacteria | 0 | 0.95161 | 0 | 0.93651 | 0.47581 |
|  | SM_Bacteria | 0.2857 | 0.8814 | 0.2222 | 0.9123 | 0.5835 |
|  | WXC_Bacteria | 0 | 0.90476 | 0 | 0.95 | 0.45238 |
|  | WYC_Bacteria | 0.25 | 0.98387 | 0.5 | 0.95312 | 0.61694 |
|  | XHD_Bacteria | 0 | 1 | NA | 0.9697 | 0.5 |
|  | XS_Bacteria | 0 | 1 | NA | 0.98485 | 0.5 |
|  | YZSM_Bacteria | 0.33333 | 1 | 1 | 0.96923 | 0.66667 |
|  | ZHCB_Bacteria | 0 | 0.98413 | 0 | 0.95385 | 0.49206 |
|  | ZHCN_Bacteria | 0.5 | 0.96875 | 0.33333 | 0.98413 | 0.73438 |
|  | ZM_Bacteria | 1 | 0.70312 | 0.09524 | 1 | 0.85156 |
|  | CT_Fungi | 0 | 1 | NA | 0.93939 | 0.5 |
|  | HLWD_Fungi | 0 | 0.87302 | 0 | 0.94828 | 0.43651 |
|  | JMWD_Fungi | 0 | 0.98333 | 0 | 0.90769 | 0.49167 |
|  | JZ_Fungi | 0.5 | 0.89062 | 0.125 | 0.98276 | 0.69531 |
|  | LBS_Fungi | 0.5 | 0.96774 | 0.5 | 0.96774 | 0.73387 |
|  | LDH_Fungi | 0 | 0.98333 | 0 | 0.90769 | 0.49167 |
|  | LHC_Fungi | 0 | 1 | NA | 0.98485 | 0.5 |
|  | LY_Fungi | 0 | 1 | NA | 0.98485 | 0.5 |
|  | MS_Fungi | 0.25 | 0.96774 | 0.33333 | 0.95238 | 0.60887 |
|  | PJ_Fungi | 0 | 0.95238 | 0 | 0.95238 | 0.47619 |
|  | RJ_Fungi | 1 | 0.98438 | 0.66667 | 1 | 0.99219 |
|  | SM_Fungi | 0.55556 | 0.96491 | 0.71429 | 0.9322 | 0.76023 |
|  | WXC_Fungi | 0.33333 | 0.85714 | 0.1 | 0.96429 | 0.59524 |
|  | WYC_Fungi | 0 | 0.93548 | 0 | 0.93548 | 0.46774 |
|  | XHD_Fungi | 0 | 1 | NA | 0.9697 | 0.5 |
|  | XS_Fungi | NA | 0.98485 | NA | NA | NA |
|  | YZSM_Fungi | 0 | 1 | NA | 0.98485 | 0.5 |
|  | ZHCB_Fungi | 0 | 1 | NA | 0.95455 | 0.5 |
|  | ZHCN_Fungi | 0.33333 | 0.95238 | 0.25 | 0.96774 | 0.64286 |
|  | ZM_Fungi | 0.8 | 0.91803 | 0.44444 | 0.98246 | 0.85902 |
