# Supplementary material for: Dynamics of Microbial Community and Potential Microbial Pollutants in Shopping Malls
Source: mSystems. 2023 Jan 5;8(1):e00576-22. doi: 10.1128/msystems.00576-22 (PMC9948725; doi:10.1128/msystems.00576-22)
Supplement: TEXT S1 [file msystems.00576-22-s0001.docx]

**Supplementary Information for**

**Dynamics of microbial community and potential microbial pollutants in shopping malls**

Xin-Li An^a^, Jian-Xin Xu^a^, Mei-Rong Xu^a,c^, Cai-Xia Zhao^a,c^, Hu Li^a^, Yong-Guan Zhu^a,b^, Jian-Qiang Su^a^#

^a^Key Laboratory of Urban Environment and Health, Institute of Urban Environment, Chinese Academy of Sciences, 1799 Jimei Road, Xiamen 361021, China

^b^State Key Lab of Urban and Regional Ecology, Research Center for Eco-environmental Sciences, Chinese Academy of Sciences, Beijing 100085, China

^c^College of Resource and Environmental Science, Fujian Agriculture and Forestry University, Fuzhou 350002, China

**Running title**: microbial community and microbial pollutants in malls

# Address correspondence to Jian-Qiang Su, [jqsu@iue.ac.cn](mailto:jqsu@iue.ac.cn).

Xin-Li An and Jian-Xin Xu contributed equally to this work. Author order was determined on the basis of seniority.

**Materials and Methods**

**Amplicon sequencing**

To profile bacterial communities, barcoded primers ArBa515F (5’-GTGCCAGCMGCCGCGGTAA-3’) and Arch806R (5’-GGACTACVSGGGTATCTAAT-3’) and FastPfu Polymerase (Miozyme) were applied to amplify the 16S rRNA genes. Fungal ITS amplicons were generated with the barcoded primers ITS1F (5’-CTTGGTCATTTAGAGGAAGTAA-3’) and ITS2R (5’-GCTGCGTTCTTCATCGATGC-3’) and rTaq Polymerase (Takara). The cycling condition was initial denaturation at 95 °C for 3 min, followed by 30 cycles of denaturing at 95 °C for 30 s, annealing at 55 °C for 30 s and elongation at 72 °C for 45 s, followed by a final elongation step at 72 °C for 10 min. Amplicons were purified using a Universal DNA Purification Kit (Tiangen Biotech Co.) and quantified using a Qubit 3.0. Purified PCR products were pooled in equimolar concentrations for constructing DNA libraries. Amplicon sequencing was performed on an Illumina MiSeq PE250 sequencing instrument (Shanghai Majorbio).

**HT-qPCR assays for human pathogens**

The SmartChip platform enables 5184-nanowell reactions per run, and HT-qPCR assay is conducted in a 100-nL reaction system. Amplification system included 1 × TaqMan Gene Expression Master Mix (Applied Biosystems, USA), DNA (5 ng/μL), forward and reverse primers (0.9 μM), probe (0.25 μM), 1 × ROX (6-carboxyl-X-rhodamine) reference dye (Invitrogen, USA), bovine serum ampere (1 mg/mL, Sigma, USA), and nuclease-free water^[1]^. The amplification conditions were set as following: 50 ℃ for 2 min and 95 ℃ for 10 min, followed by 40 cycles of 95 ℃ for 15 s and 60 ℃ for 1 min. Ten-fold serial dilutions of a composite standard plasmid mixture were made to construct the standard curves.

HT-qPCR data were analyzed using SmartChip qPCR software (V2.7.0.1). Reactions were removed when amplification efficiency was beyond the range (90%-110%) or melt curve had multiple peaks. A cutoff of cycle threshold (Ct) was set to be 31, and a marker gene would be considered a true positive only if all three technical replicates were truly positive. One pathogen would be considered to be truly positive if any of its marker genes was positively detectable. Abundance of marker genes was calculated by dividing copies of 16S rRNA gene according to standard curves.

**Identification for the Enterobacteriaceae isolates from malls**

Identification of the Enterobacteriaceae isolates was performed by sequencing the full-length 16S rRNA genes, which were amplified using the universal primers 27F (5′-AGAGTTTGATCCTGGCTCAG-3′) and 1492R (5′-TACGGYTACCTTGTTACGACTT-3′). A 25 µL PCR reaction mixture contained 1 µL of bacterial culture, 12.5µL of premix ExTaq polymerase (TAKARA, Japan) and 10 µM primers. The amplification protocol for PCR reaction was: 5 min at 94 °C, 1 min at 94 °C, 30 cycles of 1 min at 55 °C and 1 min 30 s at 72 °C, and a final extension at 72 °C for 10 min. The amplicons were gel purified using a Universal DNA Purification Kit (Tiangen Biotech Co.) and then sequenced using a Sanger sequencing approach (Bioray, China). The resulting sequences were assembled and the primer sequences were discarded. Then, the assembled sequences were annotated with the similarity ≥ 99% by BLASTn against SILVA database (version 132).

**Neutral community models for** **microbial community assembly**

To determine microbial community assembly process in shopping malls, we applied a neutral community model (NCM) to estimate the importance of stochastic processes on community assembly. The neutral community model (NCM) is based on a null hypothesis with the assumption of ‘neutral’ in ecological fitness of species, which has been successfully applied in various environments^[2-3]^. The model predicts that more abundant taxa are more likely to be dispersed by chance and therefore present in more individuals, while less abundant taxa will be lost from individuals due to ecological drift. The analyses were performed using the ASV tables for both bacterial and fungal communities, and the null model expectation was generated by bootstrapping with 1000 replicates. In the model, the parameter *R*^2^ stands for the overall fit to the neutral model, and the parameter *Nm* is an estimate of dispersal between communities. Migration rate (*m*) data were calculated basing on the analysis of observed ASV distributions and mean relative abundances fitting to the neutral model. Higher *m* values indicate that microbial communities are less limited by dispersal. Microbial taxa occurring exclusively within the 95% neutral model confidence intervals were considered to be well-predicted by the neutral model. All computations were performed in R (v4.0.3), and R code for the NCM is available according to the study by Chen et al. (2019)^[4]^.

**Random forest models and signatures identification**

Random Forest (RF) supervised learning models were used to determine the forensic power of microbial fingerprints in predicting the shopping mall, season and habitat a sample originated from and assess which microbial taxa were most associated with different shopping malls, seasons and habitats^[5]^. Random Forest classifier (RFC) were trained in the R environment (v4.0.3) with the parameter "importance = TRUE", and ASV table was divided into training (70% of the total samples) and validation (30% of the total samples) sets. RFC was then validated by using five trials of the 10-fold cross-validation and out-of-bag (OOB) sample sets in the caret package. The importance of ASVs was estimated by using Mean Decrease Accuracy and Mean Decrease Gini. Top 10 important ASVs were selected as the optimal set and RF model was constructed basing on the optimal number of predicted variables in each decision tree. The receiver operating characteristic (ROC) was constructed using pROC package to evaluate the constructed models. OOB error and accuracy (1-OOB) were calculated as a more robust estimate of generalization error, which could predict the class of a sample using a bootstrap training set without the particular sample. The variation of predicted variables basing on proximity matrix was displayed using the function MDSplot^[6]^.

**References**

1. An XL, Wang JY, Pu Q, Li H, Pan T, Li HQ, Pan FX, Su JQ. 2020. High-throughput diagnosis of human pathogens and fecal contamination in marine recreational water. Environ Res 190: 109982.

2. Burns AR, Stephens WZ, Stagaman K, Wong S, Rawls JF, Guillemin K, Bohannan BJ. 2016. Contribution of neutral processes to the assembly of gut microbial communities in the zebrafish over host development. ISME J 10: 655-664.

3. Chen QL, Hu HW, Yan ZZ, Li CY, Nguyen BAT, Sun AQ, Zhu YG, He JZ. 2021. Deterministic selection dominates microbial community assembly in termite mounds. Soil Boil Biochem 152: 108073.

4. Chen WD, Ren KX, Isabwe A, Chen HH, Liu M, Yang J. 2019. Stochastic processes shape microeukaryotic community assembly in a subtropical river across wet and dry seasons. Microbiome **7**: 138.

5. Breiman L. 2001. Random forests. Mach Learn. 1: 5-32.

6. Ren ZG, Fan YJ, Li A. 2020. Alterations of the human gut microbiome in chronic kidney disease. Adv Sci **7**: 2001936.
